# Structural insight on assembly-line catalysis in terpene biosynthesis

**DOI:** 10.1038/s41467-021-23589-9

**Published:** 2021-06-09

**Authors:** Jacque L. Faylo, Trevor van Eeuwen, Hee Jong Kim, Jose J. Gorbea Colón, Benjamin A. Garcia, Kenji Murakami, David W. Christianson

**Affiliations:** 1 Roy and Diana Vagelos Laboratories, Department of Chemistry, University of Pennsylvania, Philadelphia, PA United States; 2 Department of Biochemistry and Biophysics, Perelman School of Medicine, University of Pennsylvania, Philadelphia, PA United States; 3 Biochemistry and Molecular Biophysics Graduate Group, Perelman School of Medicine, University of Pennsylvania, Philadelphia, PA United States

**Keywords:** Enzyme mechanisms, Multienzyme complexes, Cryoelectron microscopy

## Abstract

Fusicoccadiene synthase from *Phomopsis amygdali* (PaFS) is a unique bifunctional terpenoid synthase that catalyzes the first two steps in the biosynthesis of the diterpene glycoside Fusicoccin A, a mediator of 14-3-3 protein interactions. The prenyltransferase domain of PaFS generates geranylgeranyl diphosphate, which the cyclase domain then utilizes to generate fusicoccadiene, the tricyclic hydrocarbon skeleton of Fusicoccin A. Here, we use cryo-electron microscopy to show that the structure of full-length PaFS consists of a central octameric core of prenyltransferase domains, with the eight cyclase domains radiating outward via flexible linker segments in variable splayed-out positions. Cryo-electron microscopy and chemical crosslinking experiments additionally show that compact conformations can be achieved in which cyclase domains are more closely associated with the prenyltransferase core. This structural analysis provides a framework for understanding substrate channeling, since most of the geranylgeranyl diphosphate generated by the prenyltransferase domains remains on the enzyme for cyclization to form fusicoccadiene.

## Abstract (web summary)

Substrate channeling can improve biosynthetic efficiency and has been implicated in the reactions of fusicoccadiene synthase. Here, the authors analyze this bifunctional enzyme complex by cryoEM, cross-linking MS and integrative modeling, providing structural insights into how substrate channeling is achieved.

## Introduction

The efficiency of a biosynthetic pathway can be enhanced by the covalent or noncovalent association of enzymes that catalyze sequential reactions, particularly, if the product generated by one enzyme is channeled to the active site of the next^1–4^. Substrate channeling can occur through a well-defined tunnel or instead can result simply from the proximity of one enzyme active site to the next in static or dynamic protein–protein assemblies^5,6^. Channeling does not necessarily establish kinetic superiority but instead controls the fate of a biosynthetic intermediate by minimizing diffusion into bulk solution^6^. Thus, channeling minimizes competition for biosynthetic intermediates with alternative pathways, thereby enhancing biosynthetic flux and efficiency.

Although the potential for substrate channeling through a molecular tunnel is readily determined by structural analysis, the potential for proximity channeling can be more difficult to ascertain. In the absence of driving forces such as electrostatic steering^7–9^, computational modeling suggests that the Brownian dynamics of substrate diffusion overcome the potential catalytic advantage of proximity when colocalized active sites are separated by >10 Å^10^. Even so, it appears that agglomeration of multiple enzymes can enhance proximity channeling through a probabilistic phenomenon known as cluster channeling^11^.

A unique example of substrate channeling facilitated by domain architecture and oligomerization may possibly be found in terpenoid biosynthesis^12^. Terpenoid synthases catalyze the most complex reactions in nature, in that more than half of the substrate carbon atoms undergo changes in bonding and hybridization during a single enzyme-catalyzed reaction^13–16^. The first step of terpenoid biosynthesis involves the generation of a linear isoprenoid such as C_20_ geranylgeranyl diphosphate (GGPP) from the C_5_ precursors dimethylallyl diphosphate (DMAPP) and isopentenyl diphosphate (IPP). The second step is the cyclization of the linear isoprenoid to form a polycyclic product with structural and stereochemical precision. In some cases, the catalysis of these two steps is linked in a bifunctional assembly-line terpenoid synthase.

**Fig. 1:**
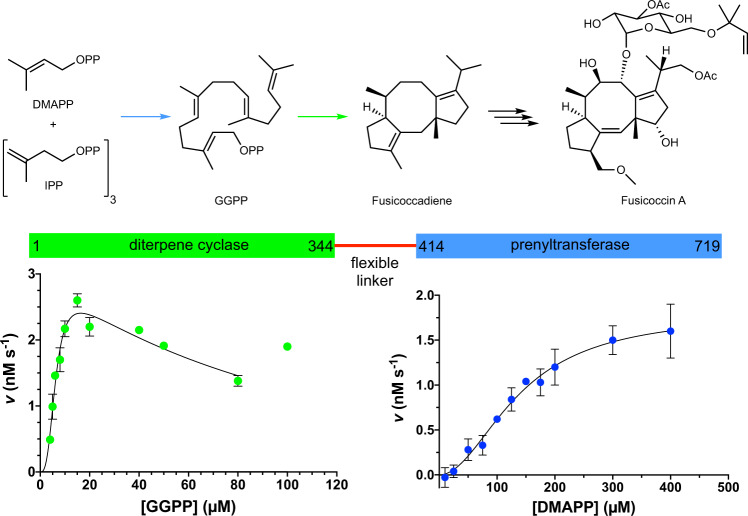
Mechanism and catalytic activity of fusicoccadiene synthase (PaFS). Prenyltransferase and diterpene cyclization reactions in the first steps of Fusicoccin A biosynthesis catalyzed by PaFS, a bifunctional assembly-line terpenoid synthase (*OPP* diphosphate, *DMAPP* dimethylallyl diphosphate, *IPP* isopentenyl diphosphate, *GGPP* geranylgeranyl diphosphate). Reaction arrow colors correspond to catalytic domain colors in the simplified representation of primary structure (blue for prenyltransferase and green for cyclase); catalytic domains are indicated by first and last residue numbers. Reaction kinetics (enzyme velocity, *v*) for each catalytic domain are also shown. The prenyltransferase reaction with DMAPP and IPP (blue data points) exhibits sigmoidal dependence on DMAPP concentrations with excellent curve fit (*R*^2^ = 0.95) and steady-state parameters *k*_cat_ = 1.01 ± 0.09 × 10^−3^ s^−1^, *K*_M_ = 139 ± 16 µM, *k*_cat_/*K*_M_ = 7.3 ± 1.5 M^−1^ s^−1^, and Hill coefficient *n* = 2.0 ± 0.3. Each data point represents the mean ±standard deviation (*n* = 3). The GGPP cyclization reaction (green data points) exhibits sigmoidal dependence on GGPP concentration as well as substrate inhibition. Taking cooperativity as well as substrate inhibition into account, data with [GGPP] ≤ 80 µM were fit to Eq. 1 (Methods), yielding an excellent curve fit (*R*^2^ = 0.93) and steady-state parameters *k*_cat_ = 0.059 ± 0.004 s^−1^, *K*_M_ = 6.4 ± 0.4 µM, *K*_i_ = 290 ± 60 µM, *k*_cat_/*K*_M_ = 9300 ± 1100 M^−1^ s^−1^, and Hill coefficient *n* = 3.0 ± 0.4. The outlier data point at [GGPP] = 100 µM is the average of two measurements and was excluded from the calculation. Each data point represents the mean ±standard deviation (*n* = 3; for [GGPP] = 20 µM, *n* = 4). Source data are provided as a Source Data file.

Consider fusicoccadiene synthase from *Phomopsis amygdali* (PaFS), a bifunctional diterpene synthase that catalyzes the first two steps in the biosynthesis of Fusicoccin A (Fig. 1)^17^. This diterpene glycoside mediates protein–protein interactions involving the 14-3-3 protein^18–20^ and stimulates axon growth and regeneration by stabilizing the 14-3-3 complex with GCN1, thereby establishing a potential therapeutic role in the repair of central nervous system injury^21^. Each catalytic domain of PaFS adopts the characteristic α-fold of a class I terpenoid synthase with αα domain architecture^22^: the C-terminal α domain is a prenyltransferase that generates GGPP, and the N-terminal α domain is a cyclase that utilizes GGPP to form tricyclic fusicoccadiene; these domains are connected by a flexible polypeptide linker ~70 residues long. Covalent attachment of these two enzymes confers a twofold enhancement in the rate of fusicoccadiene generation from DMAPP and IPP relative to separated enzymes^22^. Intriguingly, substrate channeling is implicated in the PaFS reaction sequence, as most of the GGPP generated by the prenyltransferase remains on the enzyme for cyclization instead of diffusing into bulk solution^23^.

Although full-length PaFS is refractory to crystallization, we previously reported the X-ray crystal structures of the individual prenyltransferase and cyclase domains^22^. The prenyltransferase domain crystallized with hexameric quaternary structure with D_3_ symmetry similar to that of human GGPP synthase^24^; the cyclase domain crystallized as a dimer. Previous analytical ultracentrifugation studies of full-length PaFS suggested hexameric quaternary structure^22^. In addition, small-angle X-ray scattering (SAXS) measurements with N333A/Q336A PaFS indicated hexameric quaternary structure^22^.

Here, we use cryo-electron microscopy (cryo-EM) to investigate full-length wild-type PaFS. Oligomerization is driven by the prenyltransferase domains, which predominantly form octamers, although hexamers are also observed. Cyclase domains are tethered by flexible linker segments and radiate outward in variable splayed-out positions. Crosslinking experiments additionally suggest that compact conformations can be achieved in which the prenyltransferase and cyclase domains are more closely associated. These results suggest that substrate channeling in PaFS does not occur through a tunnel, but instead results from the proximity of multiple cyclase domains—in essence, a dynamic cluster of cyclases—surrounding the oligomeric prenyltransferase core.

## Results

### PaFS exhibits oligomeric heterogeneity

Wild-type PaFS was prepared by heterologous expression in *Escherichia coli* as summarized in the Methods section^22^. Recombinant PaFS was equilibrated at room temperature for 50 min (the time interval between protein preparation and vitrification in the cryo-EM grid) and judged 99% pure and intact based on sodium dodecyl sulfate polyacrylamide gel electrophoresis (SDS-PAGE) (Supplementary Fig. 1a). The prenyltransferase and cyclase domains of recombinant PaFS were properly folded based on catalytic activity measurements of prenyltransferase activity using substrates DMAPP and IPP, and measurements of cyclase activity using substrate GGPP (Fig. 1). Thus, PaFS is catalytically active prior to vitrification.

Native gel electrophoresis indicated oligomeric heterogeneity: dimers, tetramers, hexamers, and octamers are detected, but samples of PaFS crosslinked with glutaraldehyde reveal predominantly octamers, with a minor population of hexamers also observed (Supplementary Fig. 1b). These results suggest that quaternary structure assembly is only weakly stabilized under the conditions of the native-PAGE experiment. As human GGPP synthase similarly revealed oligomeric heterogeneity with the formation of both hexamers and octamers in solution^25^, it is not unreasonable to expect similar behavior for the GGPP synthase (i.e., the prenyltransferase) domains of PaFS if these domains drive oligomerization of the full-length enzyme. Active site structures in hexamers and octamers are essentially identical (see below) and are expected to exhibit similar catalytic activity.

### Structure of PaFS indicates mobile cyclase domains

Initial two-dimensional (2D) class averages calculated from cryo-EM micrographs of full-length wild-type unliganded PaFS indicated a mixture of apparent tetramers and trimers with an approximate ratio of 90:10, largely consistent with results from negative stain EM (Supplementary Fig. 2). To our surprise, three-dimensional (3D) reconstructions from cryo-EM data clearly indicated that the tetrameric particles were in fact octamers of just prenyltransferase domains.

**Fig. 2:**
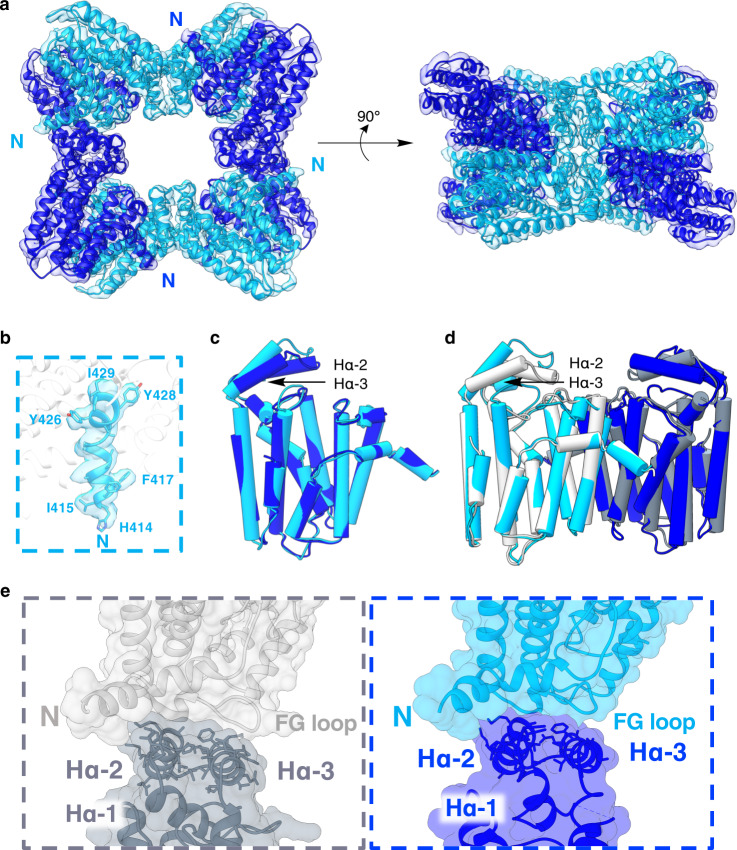
Structure of the PaFS prenyltransferase octamer. **a** Cryo-EM reconstruction of the octamer fit with the atomic model. Monomers A/A’ and B/B’ are blue and cyan, respectively, and N-termini are indicated (density map contoured at 0.141 using Chimera). **b** Density is absent prior to H414 in all monomers, as represented here by the N-terminus of chain B (map resolution range 3.69–5.12 Å, contoured at 0.0755 using Chimera). **c** Least-squares superposition of monomers A and B (rmsd = 1.5 Å for 289 Cα atoms) reveals conformational differences in capping helices Hα-2 and Hα-3. **d** The prenyltransferase AB dimer in the octamer (blue/cyan) superimposes with the corresponding dimer in the crystal structure of the hexamer (PDB 5ERN; gray) with an rmsd of 2.6 Å for 565 Cα atoms. Conformational differences are observed in capping helices Hα-2 and Hα-3. **e** Capping helices Hα-2 and Hα-3 maintain similar packing interactions with the adjacent monomer in the hexamer (left) and the octamer (right), although conformational differences in the FG loop are evident. Molecular surfaces calculated with Chimera.

The cryo-EM structure of the prenyltransferase octamer was solved to 3.99 Å resolution (Fig. 2a, Supplementary Fig. 3). Each prenyltransferase domain consists of residues 414–718. The workflow of the structure determination is summarized in Supplementary Fig. 3a and data collection and refinement statistics are recorded in Supplementary Table 1. Fourier shell correlation plots are found in Supplementary Fig. 3b, angular distribution spherical plots are shown in Supplementary Fig. 3c, and local resolution maps are displayed in Supplementary Fig. 3d. Additional maps illustrating the general quality of the structure are found in Supplementary Fig. 4a. Density for the N-terminus of each monomer ends at H414 (Fig. 2b).

The overall molecular symmetry of the octamer appears to be D_4_, but subtle structural differences confer overall C_2_ symmetry (Supplementary Fig. 4b,c). Monomers A and A’ are structurally quite similar, related by a root-mean-square deviation (rmsd) of 0.5 Å for 294 Cα atoms; the same holds true for monomers B and B’, related by an rmsd of 0.4 Å for 290 Cα atoms. However, the Hα-2 and Hα-3 helices that partially cap the active site differ in monomers A/A’ compared with monomers B/B’ (Fig. 2c). Consequently, the rmsd between monomers A and B is 1.5 Å for 289 Cα atoms, and the rmsd between monomers A’ and B’ is 1.5 Å for 288 Cα atoms. The AB dimer is superimposable with the A’B’ dimer with an rmsd of 0.5 Å for 584 Cα atoms. Thus, the octamer can be described as a tetramer of dimers. All active sites open inward to the central pore of the octamer, so substrate access to and product dissociation from the prenyltransferase domains is achieved exclusively through the central pore.

The AB dimer is superimposable with the corresponding dimer in the crystal structure of the PaFS prenyltransferase hexamer (PDB 5ERN)^22^ with an rmsd of 2.6 Å for 566 Cα atoms (Fig. 2d). Superposition of monomer A in the octamer with monomer A of the hexamer yields an rmsd of 2.0 Å for 284 Cα atoms. The more notable structural differences are mainly the Hα-2 and Hα-3 helices that partially cap the active site, which pivot outward in the octamer compared with the hexamer (Fig. 2d). In both the octamer and the hexamer, the Hα-2 and Hα-3 helices interact similarly with the N-terminal helix region of the adjacent monomer, where conformational differences in the FG loop are also observed (Fig. 2e). The Hα-2 and Hα-3 helices appear to comprise a “hinge” that readily accommodates either quaternary structure. Helices surrounding each active site in the AB dimer superimpose well in both the octamer and the hexamer (Fig. 2d), so the active site contour that serves as the template for catalysis is preserved in each oligomer.

Initial 2D class averages of the apparent trimer suggested that it was a hexamer of prenyltransferase domains assembled with D_3_ symmetry, based on the projection down the threefold symmetry axis (Supplementary Fig. 2e). This projection was identical to that of the crystal structure of the PaFS prenyltransferase hexamer (Supplementary Fig. 5). Notably, the structure of this hexamer is generally identical to that of the human GGPP synthase hexamer^22,24^. Each is a trimer of dimers, in which all active sites open inward to the central pore. The angle of one dimer to another is 30° smaller in the hexamer compared with the octamer, which is accommodated by structural differences in capping helices Hα-2 and Hα-3 (Fig. 2d) as well as differences in the contact surface between dimers (Supplementary Fig. 6). Here, too, substrate access to and product dissociation from the prenyltransferase domains are achieved exclusively through the central pore. A satisfactory 3D reconstruction of the PaFS prenyltransferase hexamer from cryo-EM data could not be achieved due to an insufficient number of particles. If the octamer and the hexamer are in thermodynamic equilibrium in solution, the 90:10 ratio observed in EM and cryo-EM studies suggests a Gibbs free energy difference ΔG of only 1.3 kcal mol^−1^ between these two oligomeric species.

Curiously, the cyclase domains are not apparent in 2D class averages and hence are not resolved in 3D reconstructions of the prenyltransferase octamer. The crystal structures of the prenyltransferase domain and the cyclase domain are sufficiently different that they can be conclusively distinguished from one another. In particular, the distinctive Hα-2 and Hα-3 helices that cap the active site of the prenyltransferase are absent in the cyclase (Supplementary Fig. 7).^22^

Where, then, are the eight cyclase domains in the full-length PaFS octamer? They are clearly present and properly folded based on our stability and catalytic activity measurements. We returned to the raw negative stain EM images of PaFS, where a closer inspection revealed satellites surrounding the central prenyltransferase particles (Supplementary Fig. 2a). Inspection of sample micrographs indicated that the more clearly visible satellites exhibited were, on average, 114 ± 28 Å away from the central prenyltransferase octamer (centroid–centroid distance), ranging from a minimum of 54 Å to a maximum of 196 Å (Supplementary Fig. 8). However, these satellites were not uniformly positioned around the octamer. These satellites, corresponding to the PaFS cyclase domains, are unresolved in class averages and cryo-EM reconstructions due to their variable positioning when the oligomer is flash-cooled. Variable positioning is a consequence of the flexibility of the 70-residue linker connecting the cyclase domain to the prenyltransferase domain. In a fully extended conformation, a 70-residue peptide segment would be ~242 Å long, and in an α-helical conformation, it would be ~104 Å long. Considering the approximate radii of the prenyltransferase octamer and individual cyclase domains, the average separation of 114 ± 28 Å suggests that a more condensed yet disordered conformation is adopted by the linker between the two domains. The wide range of distances observed (Supplementary Fig. 8b) indicates that the linker samples vast conformational space such that each cyclase domain can be separated from its cognate prenyltransferase domain by as much as 196 Å (centroid–centroid distance).

The flexible linker of PaFS corresponds to an intrinsically disordered segment, the conformational preferences of which can be broadly classified into distinct states^26^, namely, globular or extended coil^27^. The conformational preference of an intrinsically disordered polypeptide is determined by its amino-acid sequence and composition, specifically by the fraction of charged residues and the distribution of oppositely charged residues. The linker segment of PaFS corresponds to residues 345–413; analysis of this polypeptide segment using localCIDER^27^ predicts a Janus sequence, which is a classification at the boundary of globules and strong polyampholytes. Other bifunctional terpene synthases with αα domain architecture similarly contain Janus sequences for interdomain linkers (Supplementary Fig. 9). As such, the linker conformation is context-dependent and it can adopt either a condensed or extended conformation. This is consistent with our negative stain EM data and the broad distribution of prenyltransferase-cyclase separations (Supplementary Fig. 8).

### Structure of crosslinked PaFS reveals cyclase domains

**Fig. 3:**
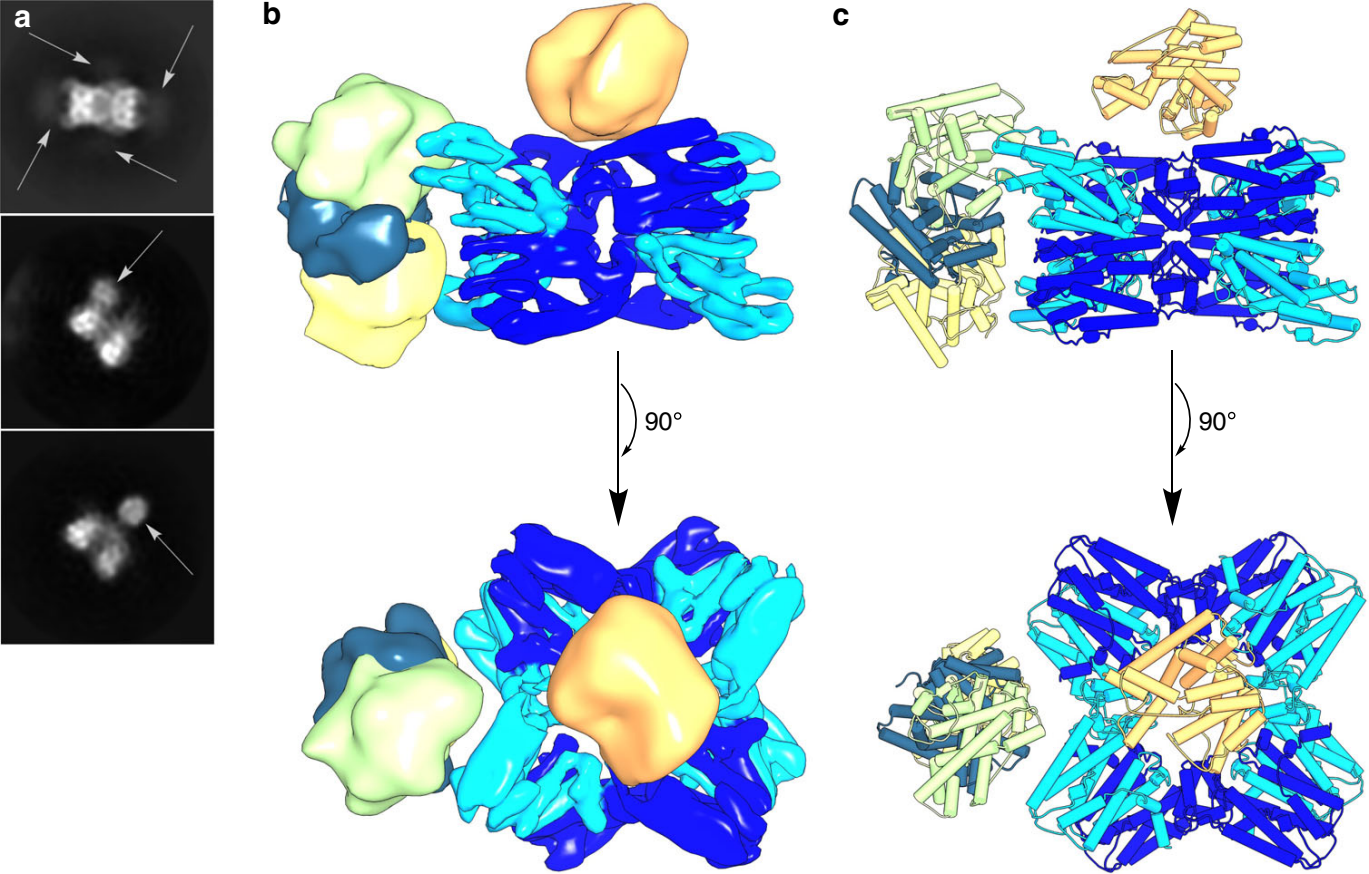
Single cyclase domains observed by cryo-EM. **a** Selected 2D class averages of fixed PaFS. Satellite domains are indicated by arrows. **b** Superimposed reconstructions of cyclase domains. Densities of the cyclase domain capping the central pore (orange, 11.9 Å resolution, contour 0.0191 in Chimera), symmetry-expanded (SE) class A (green, 8.5 Å resolution, contour 0.0132 in Chimera), SE class B (steel, 8.6 Å resolution, contour 0.0172 in Chimera), and SE class C (yellow, 9.4 Å resolution, contour 0.0203 in Chimera) were segmented from their respective full maps and superimposed with the final 7.4 Å resolution map (combining SE classes A–C) displaying prenyltransferase domains (blue and deep sky blue, contour 0.040 in Chimera). Cyclase domains are less well-resolved compared to the octameric core and do not appear at the contour displayed in the pooled map. **c** Atomic models of prenyltransferase octamer and cyclase domains (PDB 5ER8, chain A) tentatively docked into their respective maps by best fit using Chimera.

Some 2D class averages of negative stain EM images of PaFS reveal a prominent satellite that appears to correspond to at least one cyclase domain more closely associated with the central prenyltransferase octamer (Supplementary Fig. 2b). Intrigued by the indication of substrate channeling in PaFS despite the general lack of proximity between the prenyltransferase and splayed-out cyclase domains, we sought to explore how cyclase domains might associate more closely with the central prenyltransferase octamer, even if not all eight cyclase domains do so simultaneously. To this end, we employed glycerol gradient centrifugation combined with crosslinking fixation (GraFix) using glutaraldehyde^28^ for further cryo-EM studies of PaFS. Glutaraldehyde is a di-aldehyde ~4 Å long that can form imine linkages with lysine side chains on the protein surface; longer crosslinks can also be formed with polymeric glutaraldehyde di-aldehydes^29^. Not only did these data yield the familiar 2D classes of the octameric core, but density for variably positioned satellites was also clearly observed. Different 2D classes indicated that these satellites occupy different positions adjacent to the octamer (Fig. 3a): in one position, the satellite caps the central pore of the octamer, and in other positions the satellite associates with the side of the octamer.

Initial refinements of crosslinked PaFS did not extend to high resolution, partially due to converging density surrounding the octameric prenyltransferase core. Therefore, to obtain a reconstruction of the octameric prenyltransferase core of crosslinked PaFS, partial signal subtraction and masked classification were implemented to omit density outside the prenyltransferase core. This resulted in a 7.8 Å-resolution map (C1) or a 7.4 Å-resolution map (C2) (workflow outlined in Supplementary Fig. 10).

By further implementation of partial signal subtraction and masked classification (workflow outlined in Supplementary Fig. 11), a 3D reconstruction of the prenyltransferase core with a satellite capping the central pore was achieved at 11.9 Å resolution; additionally, 3D reconstructions of the prenyltransferase core with a satellite at three different positions on the side of the octamer were achieved at resolutions of 8.5–9.4 Å (Fig. 3b–c, Supplementary Table 2). The crystal structure of a single cyclase domain can be fit into the density of each pendant satellite (Supplementary Fig. 12). Furthermore, the resulting 7.4 Å-resolution map containing combined particles from the three side-associated cyclase maps (Fig. 3b) shows the central density in which helices of the octameric prenyltransferase core are well resolved.

Although a structural comparison between the 3.99 Å-resolution structure of the native prenyltransferase octamer and the crosslinked prenyltransferase octamer is necessarily limited due to the lower map resolutions of the crosslinked enzyme, the reconstruction of the native prenyltransferase octamer readily fits into the 7.4–7.8 Å-resolution densities for the crosslinked octamer using Chimera^30^ with correlation coefficients ranging from 0.83 to 0.85 (Supplementary Fig. 13). Therefore, no substantial structural changes in the octamer appear to be triggered by crosslinking or the close association of cyclase domains.

### Mass spectrometry shows prenyltransferase-cyclase crosslinks

To confirm interactions within and between the prenyltransferase and cyclase domains, we performed crosslinking mass spectrometry (XL-MS) analysis of full-length PaFS crosslinked with disuccinimidyl dibutyric urea (DSBU) (Supplementary Fig. 14, Supplementary Table 3). This reagent is capable of crosslinking lysine residues, but can also react with the hydroxyl side chains of serine, threonine, and tyrosine^31^. Covalent crosslinks observed with this reagent imply that side-chain reactive group pairs are, or are capable of achieving, inter-residue separations of 12.5 Å. Accordingly, for a lysine–lysine crosslink with a fully extended conformation, the Cα–Cα separation would be ~28.5 Å; conformational dynamics can increase this range.

**Fig. 4:**
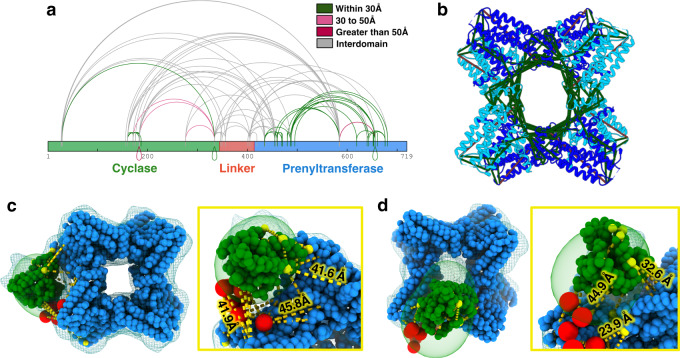
Intramolecular crosslinks observed by XL-MS. **a** Schematic representation of all observed crosslinks in full-length PaFS generated using xiView. Crosslinks are colored according to experimentally determined Cα–Cα distances: intra-cyclase interactions measured in the crystallographic dimer of the cyclase domain (PDB 5ER8) and intra-prenyltransferase interactions are measured in the cryo-EM structure of the prenyltransferase octamer. **b** Crosslinks between prenyltransferase domains in the octamer; green crosslinks indicate Cα–Cα separations < 30 Å and magenta crosslinks indicate Cα–Cα separations > 30 Å. **c** Integrative model of a single N-terminal cyclase domain and linker position relative to the prenyltransferase core. The moderate-resolution cryo-EM map of the prenyltransferase octamer with one cyclase domain (SE Class A) is displayed as blue mesh (contour 0.015), and the local probability density (LPD) of an individual cyclase domain resulting from IMP simulation is shown with a green surface. A model of the cyclase domain best fit into LPD is shown with green beads, where the X-ray crystal structure of the cyclase is represented by one residue per bead. The linker segment is modeled in red, with 10 residues per bead. Crosslinks are displayed as yellow dashed lines, with atoms harboring crosslinks displayed as yellow beads. **d** Low-resolution cryo-EM map of the prenyltransferase octamer with a capping cyclase domain displayed as blue mesh (contour 0.025), with the LPD resulting from IMP simulation of a single cyclase domain shown with a green surface. A model of the cyclase domain and linker best fit from the IMP simulation is similarly shown. The cyclase position is offset from the central pore and represents a plausible location for the cyclase between the positions observed in the cryo-EM maps shown in Fig. 3b.

Within the prenyltransferase octamer, intra- or inter-domain crosslinks revealed in this analysis are largely consistent with the observed tertiary and quaternary structure, especially within the regions of highest crosslinking density (Fig. 4a–b, Supplementary Fig. 14b, Supplementary Table 3). Crosslinks observed in the cyclase domain are also structurally consistent, taking into account that intra- or inter-molecular crosslinks involving K334 near the C-terminus can be longer than expected due to the conformational flexibility of the adjacent linker segment (Fig. 4a, Supplementary Fig. 14c, Supplementary Table 3).

Most importantly, many crosslinks are observed between the prenyltransferase domain and the cyclase domain, indicating that these domains are capable of adopting adjacent positions (Fig. 4a, Supplementary Fig. 14d). Some but not all of the prenyltransferase-cyclase crosslinks are consistent with cyclase domain positions observed in the cryo-EM structures of PaFS crosslinked with glutaraldehyde (Fig. 3, Supplementary Fig. 12). These results are consistent with a model of variably positioned cyclase domains transiently associating with the central prenyltransferase octamer.

The XL-MS data, low-resolution cryo-EM density maps, and high-resolution crystal structures of the cyclase and prenyltransferase domains of PaFS (PDB 5ER8 and 7JTH) were leveraged to build a comprehensive integrative model of the PaFS octamer using the Integrative Modeling Platform (IMP). The topology of the system was framed as a collection of nine rigid bodies: the octameric prenyltransferase core was treated as a single body and fixed during modeling, with eight satellite cyclase domains decorating the periphery of the core. In addition, a multi-scale coarse-grained representation of octamer components was prepared, summarizing highly resolved regions as single residue-per-bead particles, and regions lacking such definition through flexible coarse-grained beads each comprising up to 10 residues. Shuffling these collections of particles in space through Replica Exchange Monte Carlo (MC) simulations provided a large pool of models sampling extensive configurational space. The model pool was subjected to a scoring function evaluating the fit of each model into the cryo-EM density as well as the satisfaction of XL-MS data while taking into account the provenance ambiguity of each pair within the octameric complex (Supplementary Fig. 15). Good-scoring models, where at least 80% of observed crosslinks were satisfied within 50 Å, were considered for downstream analysis using the sampling convergence module of IMP. Models from two independent simulations were then clustered according to the positioning of the cyclase domains relative to the prenyltransferase core. This analysis resulted in a variety of structural clusters satisfying established sampling convergence criteria at precisions of ~25–35 Å; the top three structural clusters are displayed in Supplementary Fig. 15. The resulting localization probability density envelopes can be used to model cyclase domain positions relative to the prenyltransferase core, generally consistent with XL-MS and cryo-EM data (Fig. 4c–d). Although the precision of the integrative modeling is low, the results are nonetheless consistent with a model in which cyclase domains are capable of transiently associating with different positions around the octameric prenyltransferase core.

## Discussion

**Fig. 5:**
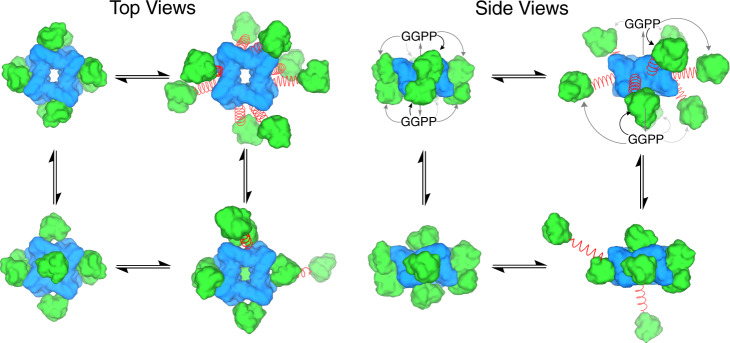
Possible conformations of the full-length PaFS octamer. Prenyltransferase domains (blue) and cyclase domains (green) are represented by molecular surfaces generated with Chimera; the flexible 70-residue linker is represented by a red spring (this color scheme corresponds to the primary structure summary in Fig. 1). Compact octamer conformations are modeled by symmetry expansion of classes A and C, class B, or capping cyclase domain positions shown in Fig. 3. Cyclase domains in splayed-out positions are modeled with an average distance of 60 Å from the prenyltransferase core. Where all cyclase domains are splayed-out, each is 60 Å from the octameric core; where only two cyclase domains are splayed-out, they are each 120 Å away. Possible trajectories of GGPP transit are illustrated in selected side view structures, in which GGPP generated by the prenyltransferase domains diffuses out of the central pore of the oligomer into the active site of one of the nearby cyclase domains.

Taken together, the negative stain EM, cryo-EM, and crosslinking data show that full-length wild-type PaFS consists predominantly of an octameric prenyltransferase core (or, less prevalent, a hexameric prenyltransferase core) with pendant cyclase domains that can adopt variable positions and orientations owing to the flexible linker connecting each cyclase to its cognate prenyltransferase. Cyclase domains can adopt positions that cap the central pore of the prenyltransferase octamer; they can adopt closely associated positions on the periphery of the octamer; or they can adopt extended, splayed-out positions with an average cyclase-octamer separation of 114 ± 28 Å (Supplementary Fig. 8). At one extreme, all cyclase domains could be associated with the octamer, and at the other extreme, all cyclase domains are splayed-out (Fig. 5). Similar conformational behavior could occur for the hexamer as well (Supplementary Fig. 16). Notably, this behavior is consistent over a wide range of protein concentrations: the PaFS concentration is 0.03 mg/mL for negative stain EM studies and 2.0 mg/mL for cryo-EM studies. Also notable is the fact that cyclase domains can associate with the central prenyltransferase octamer regardless of whether they are covalently crosslinked, based on the observation of satellite domains in EM images of native full-length PaFS (Supplementary Fig. 2).

Despite the variable positioning of the cyclase domains, competition experiments establish that most of the GGPP generated by the prenyltransferase remains on the enzyme for cyclization to form fusicoccadiene^23^. The current study shows that there is no molecular tunnel to enable GGPP transit from prenyltransferase to cyclase. Instead, the oligomerization of bifunctional protomers with cyclase satellites in constant motion around the central prenyltransferase core enables sufficient proximity and agglomeration to support dynamic cluster channeling.

The prenyltransferase releases product GGPP into the central pore of the PaFS octamer. From there, GGPP can diffuse out into one of the active sites in the cluster of eight nearby cyclase domains regardless of their precise positions (Fig. 5). If all eight cyclase domains are in motion as they surround the prenyltransferase core, dynamic cluster channeling may contribute to preferential diffusion of GGPP to the cyclase domains, perhaps facilitated through electrostatic guidance by conserved basic residues and metal ion binding^32^ in the cyclase active sites. In other words, the probability that a molecule of GGPP generated by one particular prenyltransferase domain is channeled directly to one specific cyclase domain may be low, but the probability that this GGPP will be taken up by any one of the eight surrounding cyclase domains is much higher.

Although the positions and orientations of the cyclase domains are variable, XL-MS results indicate that the cyclase domain is capable of transiently interacting with the prenyltransferase octamer (Fig. 4), and negative stain EM images suggest that stable prenyltransferase-cyclase complexes can be formed based on the appearance of satellites associated with the octamer (Supplementary Fig. 2b). Prenyltransferase-cyclase association would facilitate proximity channeling between active sites, especially when the cyclase domain caps the central pore of the prenyltransferase octamer (Fig. 3). Regardless of whether GGPP transit from prenyltransferase to cyclase is achieved by dynamic cluster channeling, proximity channeling, or a combination of both, substrate channeling in full-length PaFS does not seem to require strict control over cyclase positioning.

Prior to the discovery of PaFS as the first naturally occurring assembly-line terpene synthase,^17^ Brodelius^33^ engineered bifunctional terpene synthase constructs in which *epi*-aristolochene synthase was covalently fused with the N- or C-terminus of the prenyltransferase farnesyl diphosphate synthase to yield an approximately twofold catalytic advantage. The covalent attachment of prenyltransferase and cyclase domains in PaFS similarly gives rise to an approximately twofold catalytic advantage^22^. Although the kinetic advantage of proximity channeling is modest as it occurs in these bifunctional terpene synthases, it is important to recall that the biological advantage of substrate channeling is not necessarily rooted in kinetic superiority; instead, substrate channeling establishes more efficient carbon management to enhance biosynthetic flux^6^.

In contrast with PaFS, which is an assembly-line terpenoid synthase in which a prenyltransferase is fused with a class I cyclase with overall (αα)_8_ or (αα)_6_ architecture, copalyl diphosphate synthase from *Penicillium verruculosum* (PvCPS) is an assembly-line terpenoid synthase in which a prenyltransferase is fused with a class II cyclase with overall (αβγ)_6_ architecture^34,35^. Here, too, oligomerization is driven by the assembly of the prenyltransferase domains to form a central hexameric core, with prenyltransferase and cyclase domains in each subunit connected by a long flexible linker. Like PaFS, full-length PvCPS was refractory to crystallization, but SAXS experiments yield an overall molecular envelope with multiple, extended starburst positions for the class II cyclase domains surrounding the central prenyltransferase core^35^. It is intriguing to speculate that substrate channeling might also be operative in this and other related bifunctional terpene synthases.

The fusion of enzymes catalyzing consecutive reactions in a biosynthetic pathway to enable substrate channeling has been observed in other structurally characterized systems, most often through molecular tunnels but occasionally in the absence of a tunnel^2,4^. For example, the crystal structure of the homodimeric thymidylate synthase-dihydrofolate reductase from *Leishmania major* reveals active sites connected not by a tunnel, but by a 40 Å-long, positively charged “electrostatic highway” along the protein surface that is believed to facilitate the transfer of the negatively charged dihydrofolate intermediate from one active site to the next^7,36^. PaFS contrasts with this system by virtue of the disordered linker connecting the two catalytic domains. However, in PaFS it is conceivable that electrostatic guidance may contribute to GGPP channeling from the octameric prenyltransferase core to the surrounding cyclase domains by virtue of positively charged basic residues and metal ions at the mouth of the cyclase active site that drives substrate recognition and catalysis.

In closing, we note that structure–function relationships established for PaFS are likely representative of a new clade of bifunctional assembly-line terpenoid synthases identified in pathogenic fungi. Structure–function relationships for PaFS additionally point to catalytic strategies that can be used to guide efficient carbon management in metabolic engineering experiments^37^. Indeed, a deeper understanding of substrate channeling in terpenoid synthases may facilitate their use in synthetic biology for the efficient generation of high-value natural products for use as fragrances, cosmetics, biofuels, and drugs.

## Methods

### Protein preparation

Wild-type PaFS was expressed in *Escherichia coli* BL21-CodonPlus (DE3)-RIL (Novagen). Cells were grown at 37°C with aeration following inoculation of 6 × 1 L 2xYT medium containing 50 µg/mL kanamycin and 30 µg/mL chloramphenicol with 5 mL of starting culture. Once the optical density at 600 nm reached 0.8, cells were induced with 0.4 mM isopropyl β-d-1-thiogalactopyranoside at 16°C followed by 18 h expression. Cells were pelleted by centrifugation and resuspended in wash buffer [50 mM K_2_HPO_4_ (pH 7.5), 300 mM NaCl, 10% (v/v) glycerol, 1.5 mM tris(2-carboxyethyl)-phosphine hydrochloride (TCEP)]. After lysis by sonication, cell lysate was clarified by centrifugation and loaded on a Ni-NTA column (GE Healthcare). Protein was eluted with a 0–400 mM imidazole gradient in wash buffer. Fractions were analyzed by SDS-PAGE, and were combined and loaded onto a size-exclusion column (GE Healthcare) pre-equilibrated with 50 mM Tris (pH 7.7), 200 mM NaCl, 10% (v/v) glycerol, and 1.5 mM TCEP. The protein sample was ~99% pure as judged by SDS-PAGE. Prior to the preparation of grids for EM and cryo-EM, PaFS was dialyzed into EM buffer [2.0 mg/mL PaFS, 50 mM 4-(2-hydroxyethyl)-1-piperazineethanesulfonic acid (HEPES) (pH 7.5), 150 mM NaCl, 1.5 mM TCEP].

### Negative stain EM

PaFS samples were adsorbed onto negatively glow-discharged (1 min, easiGlow, Pelco) carbon film grids (CF200-Cu mesh, Electron Microscopy Sciences) at 0.03 mg/mL in EM buffer [50 mM HEPES (pH 7.5), 200 mM NaCl, 1.5 mM TCEP] + 10% glycerol. Samples were incubated for 30 s, washed with 2% uranyl acetate, blotted, and dried. Micrographs were collected at ×42,000 magnification on a FEI Tecnai T12 microscope operating at 120 kV and equipped with an Orius SC1000 CCD camera, with a defocus range of 0.6 to 1.5 μm and operated in low-dose mode (average dose of ~20 e/Å^2^).

Images were contrast transfer function (CTF)-corrected using CTFFIND4^38^. Particles were boxed semi-automatically using the e2boxer subroutine of the EMAN2 software suite^39^. Particles were extracted, normalized, and reference-free class averages were determined Relion (v2.1)^40^. Following iterative rounds of 2D classification, suitable class averages were used as templates for autopicking to increase the number of particles. These particles underwent further rounds of 2D classification to yield a satisfactory view of PaFS oligomer assembly (Supplementary Fig. 2), which informed expectations for cryo-EM data collection.

### Cryo-EM data collection, native PaFS

A 3 µL sample of protein solution [2.0 mg/mL PaFS, 50 mM HEPES (pH 7.5), 150 mM NaCl, 1.5 mM TCEP] was applied to glow-discharged (1 min, easiGlow, Pelco) R1.2/1.3 200-mesh copper grids (Quantifoil). Grids were blotted for 4.5 s at 100% humidity and flash-frozen with liquid ethane using a Vitrobot Mark I (FEI). Frozen grids were clipped and transferred to a Talos Arctica electron microscope (Thermo Fischer Scientific) operating at 200 keV (Pacific Northwest Cryo-EM Center). Images were recorded with a K2 direct electron detector at ×36,000 magnification (1.142 Å/pixel) at a nominal defocus of −1.5 to −2.7 µM, using SerialEM data collection software^41^. 100-frame exposures were taken at 0.1 s/frame, using a dose rate of 5.6 e^−^/pixel/s (0.43 e^−^ Å^−2^ per frame). A total of 1550 movies were recorded from one grid.

### Cryo-EM data processing, native PaFS

Data processing for native PaFS was performed using cryoSPARC (version 2.5.1)^42^. Motion correction was performed using cryoSPARC and CTF correction was performed with CTFFIND4^38^. A total of 362,627 particles were picked with the blob picker, extracted by 438.5 Å box size, and subjected to reference-free 2D classification (Supplementary Fig. 2). The same box size of 438.5 Å was used during all the downstream data analysis. To probe the possible effects of signal delocalization, particles were extracted with a box size of 384 × 384 pixels (438.5 × 438.5 Å), sufficiently large to account for density corresponding to the prenyltransferase core, linker, and eight cyclase domains. For cryoSPARC refinement, masks were automatically generated for each iteration corresponding to the calculated signal. As there was no converging density for randomly splayed-out cyclase domains outside of the prenyltransferase core, cryoSPARC consistently generated masks that excluded the signal outside of the prenyltransferase core.

The resulting 96,545 particles were used to calculate ab initio 3D reconstruction. The best 2D class averages were low-pass filtered to 20 Å and used as templates for particle picking. This resulted in 295,238 particles, which were extracted and again subjected to 2D classification to remove junk particles. With these final 94,974 particles, the initial 3D reconstruction was used as a reference to perform homogeneous refinement (without imposing symmetry), resulting in a 4.2 Å resolution map revealing eight prenyltransferase domains. CTF refinement and local motion correction were performed on particles, and without imposing symmetry subsequent refinement yielded a 4.18 Å resolution reconstruction using homogenous refinement, based on the Fourier shell correlation cutoff of 0.143. Efficiency calculations were performed in Cryo-EF^43^.

Map sharpening was achieved using DeepEMhancer^44^ (Supplementary Fig. 3). Two cryo-EM maps with and without sharpening were deposited in a single entry in the Electron Microscopy Data Bank (EMDB) with accession code EMD-23602. Upon unbiased detection of a C2 symmetry axis using proSHADE (version 0.7.5.0)^45^ (Supplementary Figure 4b), final particles were refined with C2 symmetry applied, resulting in a 3.99 Å resolution reconstruction. Map sharpening was achieved using DeepEMhancer^44^ and two cryo-EM maps with and without sharpening were deposited in a single entry in the EMDB with accession code EMD-22473. Sharpened maps with C1 and C2 symmetry are superimposable with a correlation of 0.88 determined in Chimera (Supplementary Figure 4c).

### Model building and refinement, native PaFS

Structure refinement was performed using Phenix (version dev-3736)^46^. Symmetry operators were calculated for the 3D reconstruction of PaFS with C2 symmetry using Phenix Map Symmetry. Atomic coordinates from chains A and B of the crystal structure of the PaFS prenyltransferase domain (PDB 5ERN) were docked into the 3D reconstruction using UCSF Chimera^30^. This was used as the starting model for iterative rounds of refinement and model building with Phenix Real Space Refine and Coot, respectively^46,47^. For real-space refinement, the resolution was set to 3.99 Å. Model quality was assessed with MolProbity^48^. Symmetry operators were applied to generate the final model of the octameric core using Phenix Apply NCS. Phenix comprehensive validation was used to evaluate model quality as summarized in Table S1. UCSF Chimera^30^ was used to generate figures of the model and map.

### Gradient fixation

Glycerol gradient fixation (GraFix) was performed as described by Kastner et al.^28^. In brief, PaFS (61 μM) was incubated on ice for 30 min in EM buffer [50 mM HEPES (pH 7.5), 150 mM NaCl, 1.5 mM TCEP] before ultracentrifugation. Samples were sedimented for 10 h at 4°C, 45,000 × *g* in a 10–40% glycerol gradient of EM buffer using a Beckman SW 60 Ti rotor. Glycerol gradients were prepared using a Gradient Master device (BioComp Instruments). When crosslinking was used for EM analysis, 0.125% (v/v) glutaraldehyde was added to the 40% glycerol solution before gradient preparation. Samples were then fractionated with a Piston Gradient Fractionator (BioComp Instruments). Crosslinking reactions were quenched by the addition of glycine-HCl buffer (pH 7.5) to a final concentration of 40 mM. Finally, the crosslinked PaFS complex was dialyzed into EM buffer [50 mM HEPES (pH 7.5), 150 mM NaCl, 1.5 mM TCEP] for 60 minutes to remove glycerol.

### Cryo-EM data collection, crosslinked PaFS

Samples of protein solution [3 µL of 0.3 mg/mL GraFix-stabilized PaFS, 50 mM HEPES (pH 7.5), 150 mM NaCl, 1.5 mM TCEP] were used to prepare grids in batches by manual blotting for 2.5 s using Whatman Grade 41 filter paper (Sigma-Aldrich) with a Leica EM CPC manual plunger (Leica Microsystems). Representative grids were screened on an FEI TF20 microscope operating at 200 kV and equipped with an FEI Falcon III direct electron detection camera (Electron Microscopy Resource Lab, University of Pennsylvania). Grids were imaged on a Titan Krios electron microscope (Thermo Fischer Scientific) operating at 300 keV at the National Cryo-Electron Microscopy Facility (National Cancer Institute, Frederick National Laboratory). Images were recorded with a K3 direct electron detector at ×81,000 magnification (1.08 Å/pixel) and defocus from −1.0 to −2.25 µm, using Latitude software (Gatan). Specifically, 40-frame exposures were taken at 0.08 s/frame, using a dose rate of 18.2 e^−^/pixel/s (1.25 e^−^ per Å^−2^ per frame). A total of 6544 movies were recorded from one grid.

### Cryo-EM data processing and analysis, crosslinked PaFS

For the fixed PaFS data set, motion correction (MotionCor2)^49^ and CTF Estimation (CTFFind 4.1)^38^ were performed using Relion (version 3.0.7)^50^. Particle picking was performed in cryoSPARC (version 2.15)^42^. A total of two million particles were picked with a template-based picker, extracted by a box of 384 pixels (417.7 Å) in length, and subjected to 2D classification. 2D classes showed an octamer with a clear secondary structure and neighboring flexible domains that were consistent with cyclase domains. The resulting 312,022 particles were then imported in Relion and subjected to an additional round of 2D classification, yielding 178,272 particles that were used to calculate an ab initio 3D model. 3D classification was implemented to further remove junk particles, yielding 58,496 particles with an apparent octamer similar to native unfixed PaFS. This initial reconstruction also clearly showed correlated density on the top of the octamer, i.e., capping the central pore, as well as the side of the octamer (Supplementary Fig. 11).

To resolve the prenyltransferase core, a mask was calculated from the atomic coordinates and inverted to encompass the density surrounding the prenyltransferase core. Particles were then subjected to partial signal subtraction^51^ outside the octameric core and 3D classification using the calculated mask of the prenyltransferase core (Supplementary Fig. 10). The best class contained 48,325 particles and secondary structure could be observed, consistent with non-crosslinked reconstructions. These particles were refined to 7.8 Å resolution without symmetry imposed, and to 7.4 Å resolution when imposing C2 symmetry. Reconstructions were largely consistent with non-crosslinked reconstructions (Supplementary Fig. 13). Efficiency calculations were performed in Cryo-EF^43^. The cryo-EM maps of the crosslinked PaFS prenyltransferase domain with C1 and C2 symmetry were deposited as separate entries in the EMDB (accession codes EMD-23610 and EMD-23611, respectively).

To resolve the density capping the central pore, the 58,496 particles were subjected to partial signal subtraction of the octameric core and alignment-free classification of the capping density (Supplementary Fig. 11). The best class contained 9745 particles and was consistent with a cyclase domain monomer. The corresponding 9745 particles for the entire PaFS were refined to 11.9 Å resolution, yielding an octamer with a cyclase domain consistent with positioning seen in 2D class averages (Fig. 3, Supplementary Fig. 11, Supplementary Fig. 12). To clarify the density located alongside the octamer, the 58,496 particles were subjected to symmetry expansion in Relion using C4 symmetry (Supplementary Fig. 11). Partial signal subtraction of the octameric core was performed and a mask comprised of the PaFS cyclase domain crystallographic dimer (PDB 5ER8) was used for alignment-free 3D classification. This resulted again in multiple classes with converging density corresponding to a single cyclase domain (Supplementary Fig. 11). Corresponding symmetry-expanded particles of the entire PaFS octamer and satellite domain from class A (34,712 particles), class B (34,583 particles) and class C (35,571 particles) were refined to 8.5, 8.6, and 9.4 Å resolution, respectively. Particles from classes A, B, and C were then pooled to 104,866 particles, which could then be further refined to produce a 7.4 Å map with delocalized satellite density, but with satisfactory helices observed fitting our model of the PaFS octameric core with excellent fit, 0.83, evaluated with UCSF Chimera^30^ (Fig. 3, Supplementary Fig. 11, Supplementary Fig. 12, Supplementary Fig 13). Efficiency calculations were performed in Cryo-EF^43^. The cryo-EM maps of PaFS with the capping cyclase domain, Symmetry Expanded (SE) Class A, SE Class B, SE Class C, and pooled SE classes A-C were deposited as separate entries in the EMDB (accession codes EMD-22489, EMD-22485, EMD-22487, EMD-22488, EMD-22484, respectively).

### Catalytic activity

Enzyme reaction kinetics were measured using the EnzChek™ Phosphate Assay Kit (Thermo Fisher Scientific), which enables quantitation of the inorganic pyrophosphate (PP_i_) coproduct of the prenyltransferase and cyclase reactions. Condensation of DMAPP with IPP, or cyclization of GGPP, each yield inorganic pyrophosphate (PP_i_) as a coproduct; inorganic pyrophosphatase then catalyzes the conversion of PP_i_ to inorganic phosphate (P_i_). The substrate 2-amino-6-mercapto-7-methylpurine riboside (MESG, 200 µM) is enzymatically converted by purine nucleoside phosphorylase (PNP) into ribose 1-phosphate and 2-amino-6-mercapto-7-methylpurine in the presence of P_i_, resulting in an absorbance shift from 330 nm to 360 nm.

Prenyltransferase assays were performed in triplicate on a 100-µL scale using 1.79 µM full-length PaFS in assay buffer [50 mM Tris-HCl (pH 7.5), 1 mM MgCl_2_, 100 µM sodium azide] as well as 0.002 U of PNP, MESG, and 0.00006 U of inorganic pyrophosphatase. Reactions were conducted in the presence of 600 µM IPP cosubstrate, and DMAPP concentrations ranged from 10–400 µM. Reactions were initiated by adding DMAPP substrate to a reaction mixture at 21°C. Absorbance at 360 nm was measured every 10 s using a Tecan M1000 Plate reader for 1 h, or until peak absorption was reached. Background increases in absorption were subtracted by conducting control experiments in the absence of enzyme or substrate. All measurements were made in triplicate. For each measurement, catalytic rate was determined by linear regression calculation, and converted from AU/s to nM/s using the conversion factor determined in previous study^35^; each data point represents the calculated mean rate ± standard deviation. Data were processed with Prism software and fit to an allosteric sigmoidal curve.

Cyclization assays using 50 nM PaFS were conducted in a similar manner as described for prenyltransferase assays. Substrate GGPP concentrations ranged from 4–100 µM. To enhance signal, assays using low GGPP concentrations (4–20 μM) were conducted on a 200-μL scale, in which 0.004 U of PNP and 0.00012 U of inorganic pyrophosphatase were added; remaining assays (GGPP concentration 20–100 μM) were conducted on a 100-μL scale as described for elongation assays. Reactions were initiated by adding GGPP substrate to the reaction mixture at 21°C. Absorbance at 360 nm was measured every 10 s using a Tecan M1000 Plate reader for 1 h, or until peak absorption was reached. Background changes in absorption were measured by conducting control experiments in the absence of enzyme or substrate (no increase in absorption was observed). Assays utilizing 20 μM GGPP were conducted in duplicate on a 100- and 200-μL scale, resulting in four total measurements; assay using 100 μM GGPP was conducted in duplicate due to limited substrate availability; all other measurements were made in triplicate. For each measurement, catalytic rate was determined by linear regression calculation and converted from AU/s to nM/s according to amount of PNP added; each data point represents the calculated mean rate ± standard deviation. Raw kinetic data are included in the Source Data file. Data were processed with Prism software. To account for substrate inhibition as well as cooperativity, cyclase data were fit to Eq. 1, derived from equations for allosteric sigmoidal kinetics and substrate inhibition:

1 $$\frac{{V}_{\max }\cdot {[S]}^{n}}{{{K}_{M}}^{n}+{[S]}^{n}\cdot {(1+\frac{[S]}{{K}_{i}})}^{n}}$$

Sigmoidal reaction kinetics observed for both prenyltransferase and cyclase activity (Fig. 1) imply that active sites are capable of molecular communication in PaFS oligomers. However, if pendant cyclization domains do not adopt a quaternary structure that would enable molecular communication, it is possible that this could be a system in which individual pendant cyclase monomers exhibit sigmoidal kinetics.^52^

### XL-MS sample preparation

In all, 150 μg of purified PaFS (0.5 mg/mL) in EM buffer were mixed with DSBU (final concentration of 6 mM) (Thermo Fisher Scientific) and incubated on ice for 2 h. The reaction was quenched with ammonium bicarbonate (final concentration of 50 mM). The reaction was then stopped by precipitation of crosslinked proteins with 20% (w/v) trichloroacetic acid (TCA, Sigma-Aldrich) on ice for 90 min. Proteins were sedimented by centrifugation at 21,000 × *g* for 15 min and washed with 10% TCA in 0.1 M Tris-HCl followed by acetone (Thermo Fisher Scientific). After discarding the supernatant, the pellet was air-dried and stored at -80°C for sample enrichment and preparation.

Crosslinked proteins were resuspended in 50 μl of SDS (final concentration of 2.5%) and triethylammonium bicarbonate (final concentration of 50 mM) and reduced with DTT (US Biological, final concentration of 10 mM) for 30 min at 30°C, followed by alkylation with final iodoacetamide (Sigma-Aldrich, final concentration of 50 mM) for 30 min at 30°C. To purify proteins from buffer contaminants, proteins were injected onto an S-Trap^TM^ column according to the supplier protocol (Protifi, C02-mini), and then digested with trypsin (Thermo Fisher Scientific) in 1:10 (w/w) enzyme:protein ratio for 1 h at 47°C. Peptides eluted from this column were vacuum-dried and resuspended in peptide fractionation-elution buffer [70% (v/v) LC-MS grade water (Thermo Fisher Scientific), 30% (v/v) acetonitrile (ACN, Thermo Fisher Scientific) and 0.1 % (v/v) trifluoroacetic acid (TFA, Thermo Fisher Scientific)]. For peptide fractionation, an AKTA Pure 25 was used with a Superdex 30 Increase 3.2/300 column (GE Life Science) at a flow rate of 30 μL min^−1^ with fractionation-elution buffer, collecting 100 μL fractions. Based on the elution profile, fractions containing enriched crosslinked peptides of higher molecular weights were vacuum-dried and resuspended with LC-MS grade water containing 0.1% (v/v) TFA for mass spectrometry analysis.

Analysis was performed by a Q-Exactive HF mass spectrometer (Thermo Fisher Scientific) coupled to a Dionex Ultimate 3000 UHPLC system (Thermo Fischer Scientific) equipped with an in-house made 15 cm long fused silica capillary column (75 μm ID), packed with reversed‐phase Repro‐Sil Pur C18‐AQ 2.4 μm resin (Dr. Maisch GmbH, Ammerbuch, Germany). One half of each fraction was analyzed, with elution along a gradient from 5% to 45% buffer B (90 min), followed by 90% buffer B (5 min), and re-equilibration from 90% to 5% buffer B (5 min) with a flow rate of 400 nL min^−1^ (buffer A: 0.1% formic acid in water; buffer B: 0.1% formic acid in 80% ACN). Data were acquired in data-dependent MS/MS mode, with the following full scan MS settings: mass range 300−1800 m/z, resolution 120,000; MS1 AGC target 1E6; MS1 Maximum IT 200; and the following MS/MS settings: resolution 30,000; AGC target 2E5; MS2 Maximum IT 300 ms; fragmentation enforced by higher-energy collisional dissociation with stepped collision energy of 25, 27, 30; loop count top 12; isolation window 1.5; fixed first mass 130; MS2 Minimum AGC target 800; charge exclusion: unassigned, 1, 2, 3, 8, and >8; peptide match off; exclude isotope on; dynamic exclusion 45 s. Raw files were converted to mgf format with TurboRawToMGF 2.0.8^53^.

### Crosslinked peptide search

Search engine MeroX 2.0.1.4 was used to identify and validate crosslinked peptides^54^. MeroX was run in RISEUP mode, using default crosslinker mass and fragmentation parameters for DSBU: precursor mass range 1000–10,000 Da; minimum precursor charge 4; precursor and fragment ion precisions 5.0 and 10.0 ppm, respectively; maximum number of missed cleavages 3; carbamidomethylation of cysteine and oxidation of methionine, as fixed and variable modifications, respectively. Results were filtered for score (>10) and false discovery rate, FDR ( < 1%) (Supplementary Data 1). Fasta database was constructed by adding the PaFS sequence (uniport ID) to the entire *E. coli* BL21 proteome sequence database along with the cRAP database (https://www.thegpm.org/crap/), which consists of common contaminants, and was added by MeroX. Each acquisition was searched and the FDR calculated separately through MeroX and the final result is assembled from those individual results. Crosslinks were visualized on the PaFS structure using UCSF Chimera^30^. Mass spectrometry data were deposited into the ProteomeXchange Consortium via the PRIDE repository^55^ with data set identifier PXD021007. The program xiView was used to prepare the schematic representation of all observed crosslinks (Fig. 4a)^56^.

### Integrative modeling

Integrative modeling of the PaFS homo-octamer was performed on the IMP^57,58^ software. To perform modeling, XL-MS data (Supplementary Table 3), low-resolution cryo-EM density envelopes (EMDB: EMD-22489, EMD-22485, EMD-22487, EMD-22488), and atomic structures of the N-terminal (PDB 5ERM) and C-terminal (PDB 7JTH, this study) domains accounting for 83% of the protein sequence were input into IMP (Supplementary Table 4). Additional information including sequence, backbone connectivity, and non-overlapping volume constraints were used. Cryo-EM densities were modeled using 3D Gaussian mixture models of 200 functions with satisfactory cross-correlation coefficients (Supplementary Table 4). A total of 83 XL-MS restraints derived from DSBU crosslinking experiments were used (Supplementary Table 3). These crosslinks were distributed as follows: 23 intra-cyclase domain, 27 intra-prenyltransferase domain, and 23 interdomain crosslinks, with the remaining 10 involving the linker region. Crosslinks were considered individually for each monomer in the complex weighted by its identification score (Supplementary Table 3). The system topology consisted of nine independently moving rigid bodies—one core prenyltransferase octamer and eight cyclase domains. The system was represented in coarse-grained fashion: one bead per residue where high-resolution information is available, and one bead per 10 residues for the remainder of the protein sequence. The model was also represented using 3D Gaussian functions at 10 residues per function for use during EM satisfaction score determination. MC simulations with replica exchange were carried out using a highly parallel computing system starting at 160 distinct initial configurations, sampling 5000 frames, with 10 MC steps between model output. Two independent simulations were run for each system, analyzed using IMP’s sampling convergence module and evaluated for sampling convergence based on the localization of the cyclase domains (Supplementary Table 4). Additionally, convergence was examined for subsets of good-scoring models where at least 80% of crosslinking information was satisfied at 50 Å distances. Figures were made in ChimeraX^59^. Data for the entire project including inputs, modeling scripts, analysis scripts, and results may be accessed at the following online repository: https://github.com/cryomurakami/Structural-Insight-on-Assembly-Line-Catalysis-in-TerpeneBiosynthesis.^60^

### Reporting summary

Further information on research design is available in the Nature Research Reporting Summary linked to this article.

## Supplementary information

Supplementary Information

Description of Additional Supplementary Files

Supplementary Dataset 1

Reporting Summary

## Data Availability

Atomic coordinates of the PaFS prenyltransferase octamer (C2 symmetry) have been deposited in the Protein Data Bank (www.rcsb.org) with PDB accession code 7JTH, and in the EMDB with accession code EMD-22473. The asymmetric (C1) density map has also been deposited in the EMDB with accession code EMD-23602. Additionally, density maps of the crosslinked PaFS prenyltransferase octamer have been deposited in the EMDB with accession codes EMD-23610 (C1) and EMD-23611 (C2). Density maps of the fixed PaFS prenyltransferase octamer with associated cyclase domains have been deposited in the EMDB with accession codes as follows: capping cyclase domain, EMD-22489; SE Class A domain, EMD-22485; SE Class B domain, EMD-22487; SE Class C domain, EMD-22488; Pooled SE Domain Classes A–C, EMD-22484. The common Repository of Adventitious Proteins (cRAP) database used in this study can be accessed at https://www.thegpm.org/crap. XL-MS data have been deposited in the PRIDE repository with data set identifier PRIDE: PXD021007. Previously determined crystal structures of the individual PaFS prenyltransferase and cyclase domains were also used in this study and can be accessed in the PDB with accession codes 5ERN and 5ERM or 5ER8, respectively. All other relevant data are available from the corresponding author upon request. Source data are provided with this paper.

## Source data

Source Data

## Code availability

Code to reproduce results presented in this manuscript as well as the PaFS models can be found at https://github.com/cryomurakami/Structural-Insight-on-Assembly-Line-Catalysis-in-TerpeneBiosynthesis and in the related Zenodo deposition^60^.
