# Supplementary material for: Structural insight on assembly-line catalysis in terpene biosynthesis
Source: Nat Commun. 2021 Jun 9;12:3487. doi: 10.1038/s41467-021-23589-9 (PMC8190136; doi:10.1038/s41467-021-23589-9)
Supplement: Supplementary file 1 — Supplementary Information [file 41467_2021_23589_MOESM1_ESM.pdf]

--- SUPPLEMENTARY INFORMATION ---

Structural Insight on Assembly-Line Catalysis in Terpene Biosynthesis

Jacque L. Faylo,[1] Trevor van Eeuwen,[2] Hee Jong Kim,[2] Jose J. Gorbea Colón,[2] Benjamin A. Garcia,[2] Kenji Murakami,[2] and David W. Christianson[1,*]

[1]Roy and Diana Vagelos Laboratories, Department of Chemistry, University of Pennsylvania, 231 South 34th Street, Philadelphia, PA 19104-6323, United States

[2]Department of Biochemistry and Biophysics, Perelman School of Medicine, University of Pennsylvania, Philadelphia, PA 19104-6323, United States

Supplementary Information

Supplementary Table 1. Data Collection and Refinement Statistics.

	PaFS Prenyltransferase Octamer (EMD-22473)	Asymmetric PaFS Prenyltransferase Octamer (EMD-23602)
**EM data collection and reconstruction statistics**		
Magnification	36,000x	36,000x
Voltage (kV)	200	200
Exposure (e^-/Å^2)	43	43
Defocus range (μm)	1.5 - 2.7	1.5 - 2.7
Pixel size (Å/pix)	1.142	1.142
Symmetry imposed	C2	C1
Initial particles (no.)	295,238	295,238
Final particles (no.)	94,974	94,974
Map resolution (Å)		
FSC threshold 0.143	3.99	4.18
**Model**		
Initial model used (PDB code)	5ERN (Chains A and B)	
Model composition (#)		
Chains	8	
Atoms	14628 (Hydrogens: 0)	
Residues	Protein: 2332	
Water	0	
Ligands	0	
R.m.s. deviations		
Length (Å)	0.003	
Angles (°)	0.6	
**Validation**		
MolProbity score	2.06	
Clash score	9.28	
Poor rotamers (%)	0.45	
Ramachandran plot (%)		
Favored	89.39	
Allowed	10.03	
Disallowed	0.61	
Peptide plane (%)		
Cis proline/general	0.0/0.0	
Twisted proline/general	0.0/0.0	
PDB accession code	7JTH	
**Model vs. Data**		
CC (mask)	0.74	
CC (box)	0.79	
CC (peaks)	0.76	
CC (volume)	0.76	

Supplementary Table 2. Data Collection and Refinement Statistics of Fixed PaFS.

	Crosslinked PaFS prenyl-transferase octamer (C2) (EMD-23611)	Crosslinked PaFS prenyl-transferase octamer (C1) (EMD-23610)	PaFS with capping cyclase domain (EMD-22489)	PaFS with pooled peripheral cyclase domains (EMD 22484)	PaFS with peripheral cyclase domain A (EMD-22485)	PaFS with peripheral cyclase domain B (EMD-22487)	PaFS with peripheral cyclase domain C (EMD-22488)
**Data collection and processing**							
Magnification	81,000	81,000	81,000	81,000	81,000	81,000	81,000
Voltage (kV)	300	300	300	300	300	300	300
Electron exposure ($e^-/Å^2$)	50	50	50	50	50	50	50
Defocus range (μm)	1 - 2.25	1 - 2.25	1 - 2.25	1 - 2.25	1 - 2.25	1 - 2.25	1 - 2.25
Physical pixel size (Å)	1.08	1.08	1.08	1.08	1.08	1.08	1.08
Symmetry imposed	C2	C1	C1	C4 (symmetry expanded) C1 (refinement)	C4 (symmetry expanded) C1 (refinement)	C4 (symmetry expanded) C1 (refinement)	C4 (symmetry expanded) C1 (refinement)
Initial particles (no.)	312,022	312,022	312,022	312,022	312,022	312,022	312,022
Final particles (no.)	48,325	48,325	9,745	104,866	34,712	34,583	35,571
Map resolution (Å) at FSC threshold 0.143	7.4	7.8	11.9	7.4	8.5	8.6	9.4
**Model**							
Prenyltransferase fitting model (PDB code)	7JTH						
Cyclase fitting model (PDB code)	5ER8						

Supplementary Table 3. Observed crosslinks determined by XL-MS.

Peptide 1	Peptide 2	Abs Pos 1	Abs Pos 2	Relative Score	Dist (Å)	# Meas.
**(a)**	**Prenyltransferase-Prenyltransferase Cross Links**					
KHGGMTLEQK	KHGGMTLEQK	656	656	100	5.6	4
VPDVKVGK	IKDAVR	455	460	71.0	8.4	8
SHMALLNVLSTGR	QFVLDIIEEEKSLDYTR	643	676	70.4	12.4	8
SHMALLNVLSTGR	KHGGMTLEQK	652	656	68.6	5.9	16
GKPSTHNIFGSAQTVNTATYSIIK	QFVLDIIEEEKSLDYTR	486	681	64.3	25.3	8
GKPSTHNIFGSAQTVNTATYSIIK	KHGGMTLEQK	486	656	61.2	19.0	8
QKGFBEDLDEGK	KHGGMTLEQK	619*	656	59.8	18.0	8
SHmALLNVLSTGR	KHGGMTLEQK	653	656	58.7	4.9	4
GKPSTHNIFGSAQTVNTATYSIIK	SHMALLNVLSTGR	486	643	56.4	11.9	8
WSLALIHMIHKQR	QFVLDIIEEEKSLDYTR	640	676	55.4	10.9	8
AVLEAPYDYIASMPSKGVR	GKPSTHNIFGSAQTVNTATYSIIK	435	486	50.3	15.1	8
IKDAVR	TKIQSBLHR	460	585	48.0	23.3	8
VPDVKVGK	TKIQSBLHR	455	584	47.5	18.2	8
RGKPSTHNIFGSAQTVNTATYSIIK	QFVLDIIEEEKSLDYTR	488	676	47.0	22.9	8
KHGGMTLEQK	HGGMTLEQKQFVLDIIEEEK	656	661	45.4	11.8	16
AVLEAPYDYIASMPSKGVR	VLHNSSLLLDDFQDNSPLR	435	469	43.1	16.0	8
VLHNSSLLLDDFQDNSPLRR	KHGGMTLEQK	480	656	37.9	17.8	32
GKPSTHNIFGSAQTVNTATYSIIK	WSLALIHMIHKQR	486	640	32.6	17.4	8
TKIQSBLHR	KHGGMTLEQK	585	656	31.8	50.5	0
IKDAVR	VLHNSSLLLDDFQDNSPLR	460	469	31.1	13.6	8
IKDAVR	VLHNSSLLLDDFQDNSPLRR	460	470	28.6	14.9	8
GKPSTHNIFGSAQTVNTATYSIIK	KHGGMTLEQK	495	656	24.8	13.0	24
AVLEAPYDYIASMPSKGVR	IKDAVR	434	460	24.5	13.6	8
GKPSTHNIFGSAQTVNTATYSIIK	QKGFBEDLDEGK	486	619*	18.4	17.1	10
VLHNSSLLLDDFQDNSPLRR	GKPSTHNIFGSAQTVNTATYSIIK	480	486	17.3	10.3	8

Peptide 1	Peptide 2	Abs Pos 1	Abs Pos 2	Relative Score	Dist (Å)
**(b)**	**Cyclase-Cyclase Cross Links**				
FVELAKYIPYR	FVELAKYIPYR	183	183	83.9	n/a
YNPDVSFNKTQLEWM	YNPDVSFNKTQLEWM	334	334	71.7	n/a
SKFTTLEDR	YNPDVSFNKTQLEWMR	28	334	64.3	19.1
KLIVEYVAK	YNPDVSFNKTQLEWMR	276	334	61.3	37.4
FVEVGSSR	FVELAKYIPYR	171*	183	54.8	16.7
FVELAKYIPYR	YNPDVSFNKTQLEWMR	183	334	51.1	44.0
TTMKSWAR	FVEVGSSR	160	171*	41.1	17.7
TTMKSWAR	FVELAKYIPYR	160	187	27.0	21.2
QHETR	FVELAKYIPYR	176	183	26.9	10.3
FVEVGSSR	YNPDVSFNKTQLEWMR	171*	334	24.5	33.2
TTMKSWAR	FVELAKYIPYR	160	183	14.1	27.7
**(c)**	**Cyclase-Prenyltransferase Cross-Links**				
KSKFTTLEDR	TKIQSBLHR	27	585	74.0	28.4
FVEVGSSR	TKIQSBLHR	171*	585	67.8	50.5
KLIVEYVAK	TKIQSBLHR	276	585	67.2	41.3
TKIQSBLHR-TTMKSWAR	TKIQSBLHR	160	585	65.2	26.7
TKIQSBLHR-SKFTTLEDR	TKIQSBLHR	28	585	64.9	24.6
YNPDVSFNKTQLEWMR	TKIQSBLHR	334	584	60.6	25.0
FVELAKYIPYR	VPDVKVGK	184	455	59.6	41.9
FVELAKYIPYR	TKIQSBLHR	183	584	57.3	25.0
YNPDVSFNKTQLEWMR	VPDVKVGK	334	455	56.7	41.2
FVELAKYIPYR	AVLEAPYDYIASMPSKGVR	183	435	52.5	42.2
IQSQLFLEMLAIDPEBAKTTMK	TKIQSBLHR	156	584	50.2	23.7
SKFTTLEDR	VPDVKVGK	28	455	42.7	50.9
KLIVEYVAK	VPDVKVGK	276	455	42.0	45.2
QHETR	TKIQSBLHR	176	585	41.5	19.3
TTMKSWAR	VPDVKVGK	160	455	32.5	43.3
FVELAKYIPYR	VGKIK	183	458	22.7	42.4
**(d)**	**Cross Links in Linker Region***				
YNPDVSFNKTQLEWMR	SPEIDSDESAVSPTADESDSTEDSLGSGSR	334	356	64.6	
QDSSLSTGLSLSPVHSNEGKDLQR	QFVLDIIEEEKSLDYTR	405	681	63.1	
YNPDVSFNKTQLEWMR	QGLPSLESBPVLAR	334	349	58.0	
QDSSLSTGLSLSPVHSNEGKDLQR	TKIQSBLHR	405	585	54.3	
FVELAKYIPYR	QDSSLSTGLSLSPVHSNEGKDLQR	183	405	49.1	

QDSSLSTGLSLSPVHSNEG**K**DLQR	WSLALIHMIH**K**QR	405	640	46.9
QDSSLSTGLSLSPVHSNEG**K**DLQR	**S**HMALLNVLSTGR	405	643	46.7
QDSSLSTGLSLSPVHSNEG**K**DLQR	VDTDHIFFE**K**AVLEAPYDYIASMPSK	405	419	44.2
YNPDVSFN**K**TQLEWMR	QDSSLSTGLSLSPVHSNEG**K**DLQR	334	405	41.8
QGLP**S**LESBPVLAR	T**K**IQSBLHR	346	585	41.8
KS**K**FTTLEDR	**S**PEIDSDESAVSPTADESDSTEDSLGSGSR	29	356	41.2
QGLPSLE**S**BPVLAR	QDSSLSTGLSLSPVHSNEG**K**DLQR	349	405	40.6
**S**PEIDSDESAVSPTADESDSTEDSLGSGSR	T**K**IQSBLHR	356	585	34.4
**S**PEIDSDESAVSPTADESDSTEDSLGSGSR	**K**HGGMTLEQK	356	656	30.8
QDSSLSTGLSLSPVH**S**NEGKDLQR	G**K**PSTHNIFGSAQTVNTATYSIIK	401	486	30.4
QDSSLSTGLSLSPVH**S**NEGKDLQR	VPDV**K**VGK	401	455	27.0
KS**K**FTTLEDR	QGLPSLE**S**BPVLAR	29	349	15.1
QDSSLSTGLSLSPVHSNEG**K**DLQR	I**K**DAVR	405	460	11.7

* Disordered in structure

Crosslinked amino acids are indicated in bold red letters. The absolute position numbering of each crosslink corresponds to the amino acid sequence of PaFS. Relative scores are derived from MeroX, normalized to the highest score. The FDR confidence levels derive from the MeroX search results. Raw MeroX scores, along with measured m/z, charge, experimental mass, theoretical mass, and mass error in ppm are provided in Supplementary Data 1. MeroX search details and parameters are given in the Methods. An asterisk (*) indicates that the crosslinked residue is disordered in the atomic coordinates, and the listed distance is reported from integrative modeling. (a) Observed prenyltransferase-prenyltransferase crosslinks. Crosslink distances were measured using the structural model of the prenyltransferase domain (PDB 7JTH) reported in this study, and distances between Cα atoms of identified residues are listed under the column header Dist (Å). In the octameric prenyltransferase domain, since many intermolecular interactions are possible per crosslink, the number of satisfactory distances (within 35 Å) that were measured are listed under column header # meas. (b) Observed cyclase-cyclase crosslinks. Crosslink distances were measured using the structural model of the cyclase domain (PDB 5ERM) determined by X-ray crystallography, and distances between Cα atoms of identified residues are listed under the column header Dist (Å). (c) Cyclase-prenyltransferase crosslinks. Inter-domain crosslink distances were measured using the prenyltransferase octamer model (PDB 7JTH), with a model of the cyclase domain (PDB 5ERM) docked into low-resolution cryo-EM density according to best fit in Chimera (manual fitting of domains into cryo-EM maps and measured crosslinks are shown in Supplementary Fig. 14d). Distances were calculated between Cα atoms and are listed under column header Dist (Å). (d) Crosslinks involving residues in the linker segment (residues 345-413 in the amino acid sequence).

Supplementary Table 4. Integrative modeling analysis and statistics.

Analyses		Score Distribution		Sampling Precision Statistics					Gaussian Mixture Models		
EM Map	XL-MS satisfaction cutoff (Å)	K-S test D	K-S test p-value	Number of Clusters	P-value	Cramer's V	Clustered Population (%)	Sampling Precision (Å)	Number of GMMs	Thres-hold	Chimera Cross-Correlation Coeff.
SE_A	50	0.018	1	15	0	0.097	93.1	28.4	200	0.015	0.99
SE_B	50	0.011	1	7	0	0.084	98.5	37.2	200	0.015	0.98
SE_C	50	0.017	1	8	0	0.08	96.1	34.3	200	0.015	0.99
Cap	50	0.03	0.01	11	0.015	0.047	93.1	28.3	200	0.025	0.99

Simulations were conducted with Gaussian mixture models (GMMs) generated from input EM maps and XL-MS restraints to a 50 Å distance. The Kolmogorov-Smirnov two-sample test was performed to ensure score distributions are similar if p-value > 0.05 or D < 0.30. Sampling precision was determined by three criteria: (1) p-value from χ^2 test for homogeneity of proportions > 0.05; (2) Cramer's V value for difference between the distributions in two samples < 0.10; (3) the population of clustered models after the removal of the cluster containing less than 10 models from either sample > 0.80. From those criteria, overall precision of simulation sampling ranges was estimated to range from 28 to 37 Å. GMMs from the densities were generated by the number of centers and threshold shown in the table. The models were evaluated through Chimera cross-correlation calculation.

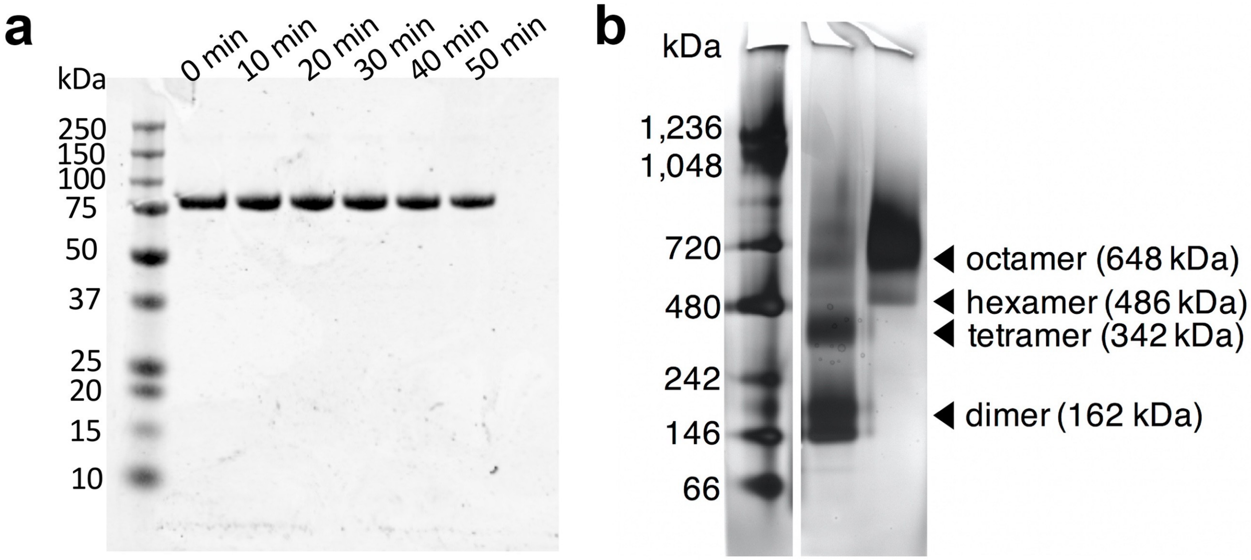

Supplementary Fig. 1. (a) Stability and activity of full-length PaFS. PaFS (2.0 mg/mL) was equilibrated at room temperature in EM buffer and 20-μg aliquots were extracted every 10 min. SDS-PAGE analysis revealed that full-length PaFS (81 kD) remained intact for at least 50 min, the time interval between protein preparation and vitrification. No evidence for sample degradation was observed, thus verifying that EM and cryo-EM grids were prepared with pure and fully-intact PaFS. This time-course evaluation was conducted once, although PaFS at 81 kD on an SDS-PAGE is highly reproducible. **(b)** Native-PAGE with glycerol gradient fixation (GraFix). PaFS (61 μM) in the presence and absence of 0.000–0.125% glutaraldehyde for fixation was subject to ultracentrifugation and fractionation (see Methods). Analysis by SDS-PAGE revealed that PaFS eluted at identical fractions both with and without glutaraldehyde, indicating that the density/molecular weight of PaFS is unchanged after cross-linking. Accordingly, identical fractions were taken from both the fixed and unfixed samples of PaFS and subjected to Native-PAGE, shown above. A total of 5 μg of PaFS (0.3 mg/mL) of native protein (lane 2) and GraFix-stabilized protein (lane 3) were run against the standards in lane 1. The non-cross-linked PaFS sample exhibits oligomeric heterogeneity, with the more prominent bands corresponding to octamers, hexamers, tetramers, and dimers. This behavior is consistent with that recently observed for a different bifunctional terpene synthase, copalyl diphosphate synthase from *Penicillium verruculosum*,[1] suggesting that quaternary structure assembly is only weakly stabilized under the conditions of the Native-PAGE experiment. The cross-linked PaFS sample exhibits predominantly octameric quaternary structure, with a minor population of hexamer also observed. This is consistent with our observations by cryo-EM, and these results have been reproduced in two other independent experiments. Source data are provided as a Source Data file.

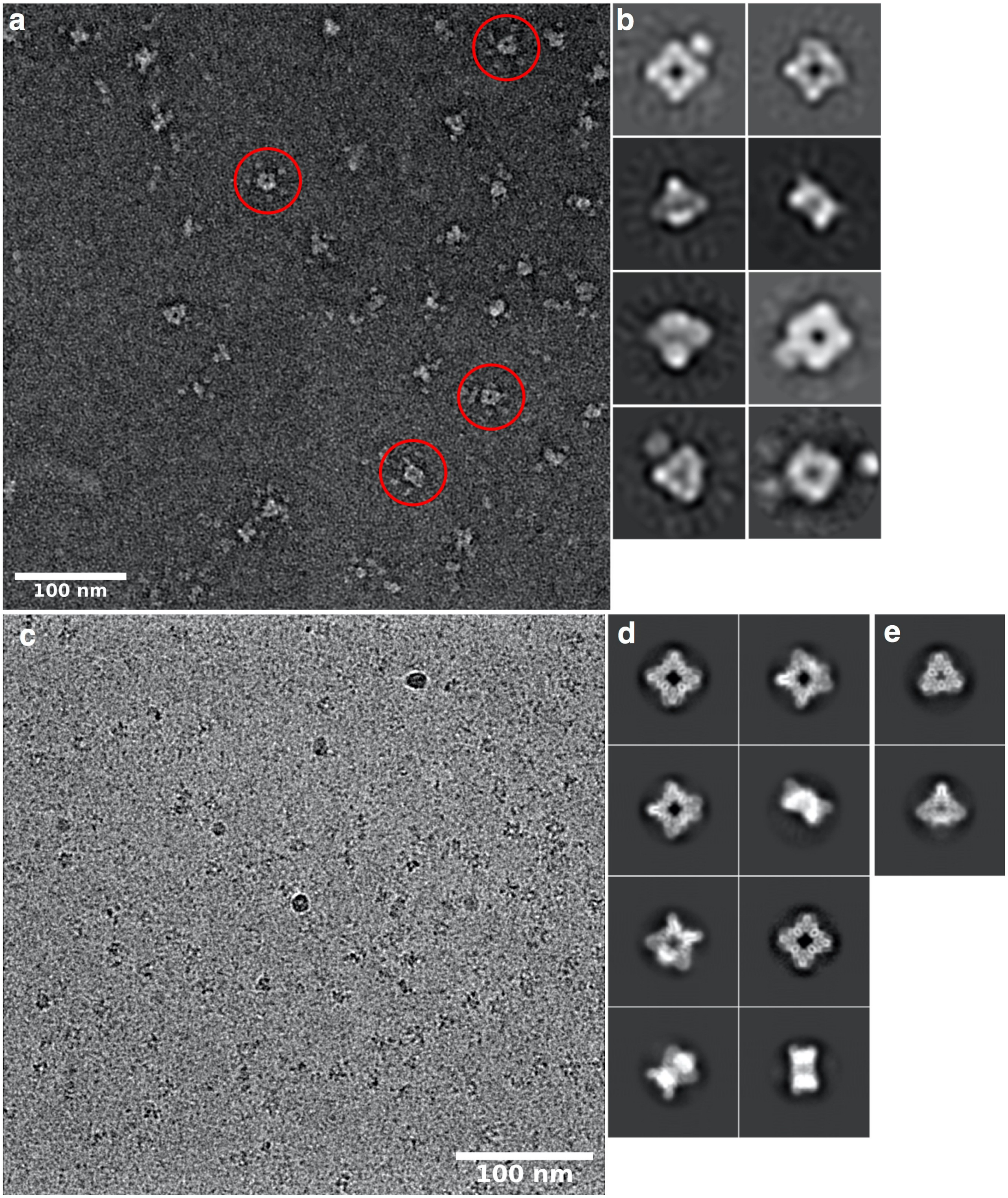

Supplementary Fig. 2. Visualizing PaFS in raw micrographs and class averages. (a) Representative negative stain electron micrograph of PaFS with scale bar indicating 100 nm. Micrograph is representative of a dataset of 102 collected micrographs from one grid; similar and reproducible results were obtained from independent preparation of two other grids. Selected satellites surrounding central octamers are highlighted by red circles. **(b)** 2D class averages of PaFS octamers and hexamers from negative stain EM data. Satellite domains are evident in some of these reconstructions. **(c)** Representative cryo-EM micrograph of PaFS with scale bar indicating 100 nm. Micrograph is representative and results are reproducible across a dataset of 1550 collected movies. **(d)** 2D class averages of the PaFS octamer from cryo-EM data. **(e)** 2D class average of the PaFS hexamer from cryo-EM data.

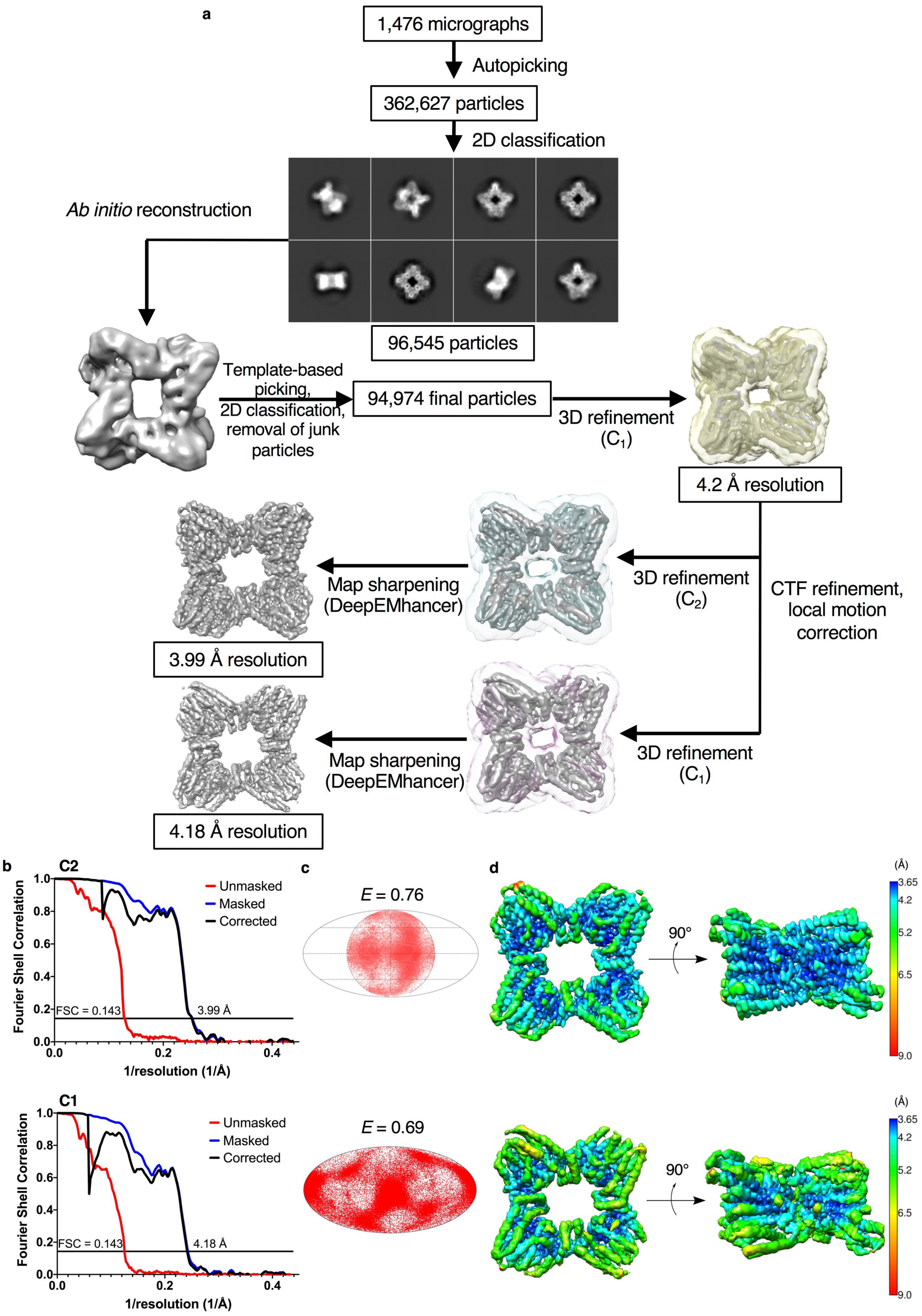
a
1,476 micrographs
Autopicking
362,627 particles
2D classification
Ab initio reconstruction
96,545 particles
Template-based picking, 2D classification, removal of junk particles
94,974 final particles
3D refinement (C_1)
4.2 Å resolution
3D refinement (C_2)
CTF refinement, local motion correction
Map sharpening (DeepEMhancer)
3.99 Å resolution
3D refinement (C_1)
Map sharpening (DeepEMhancer)
4.18 Å resolution
b
C2
Fourier Shell Correlation
Unmasked
Masked
Corrected
FSC = 0.143
3.99 Å
1/resolution (1/Å)
C1
4.18 Å
c
E = 0.76
E = 0.69
d
(Å)
3.65
4.2
5.2
6.5
9.0
90°

Supplementary Fig. 3. Workflow for cryo-EM structure determination of PaFS. (a) A total of 1,476 out of 1,550 micrographs were chosen based on motion and contrast transfer function (CTF) assessment in cryoSPARC. Autopicking, ab initio model reconstruction, 3D refinement, CTF refinement, and local motion correction were conducted in cryoSPARC. Map sharpening was performed with DeepEMhancer. **(b)** Fourier shell correlation (FSC) plots for unmasked (red), masked (blue), and corrected (black) maps calculated in cryoSPARC, for maps calculated with C2 (top) and C1 (bottom) symmetry. Resolution estimate at 0.143 FSC is highlighted for the corrected map. **(c)** Angular distribution spherical plots of data used in reconstruction of C2 (top) and C1 (bottom) maps. Efficiencies were calculated in cryo-EF and are listed as *E*. **(d)** Local resolution maps of PaFS prenyltransferase reconstructions calculated with C2 (top) and C1 (bottom) symmetry. Local resolution is depicted according to the scale shown in Å at 0.5 FSC. Maps calculated with cryoSPARC and contoured at 0.141 using Chimera.

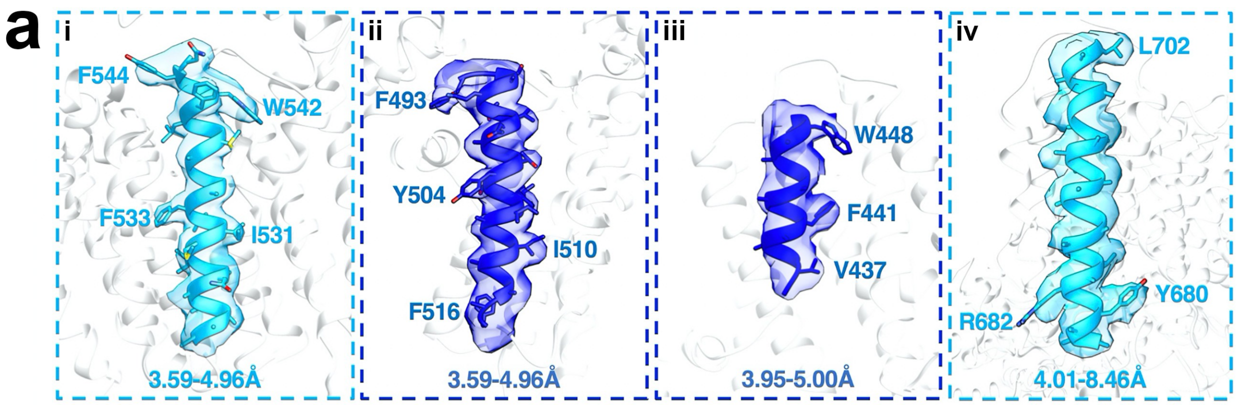

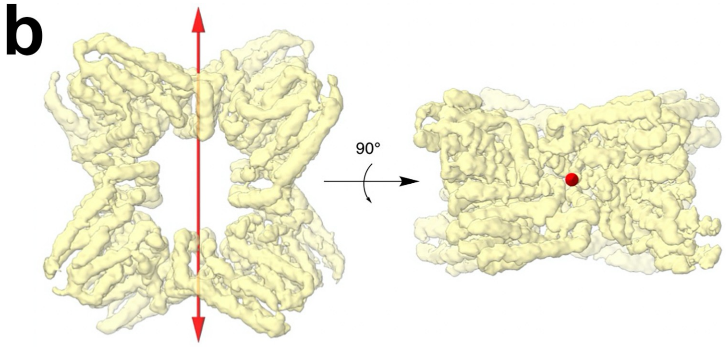

C symmetry axes

Fold	X	Y	Z	Angle	Height
**+2**	**+0.02408**	**-0.53856**	**-0.84224**	**+3.14159**	**+0.80189**
+2	+0.03440	-0.98012	-0.19539	+3.14159	+0.34644
+2	+0.00926	+0.23277	-0.97249	+3.14159	+0.32982

D symmetry axes

Fold	X	Y	Z	Angle	Height
+2	+0.03440	-0.98012	-0.19539	+3.14159	+0.34644
+2	+0.00926	+0.23277	-0.97249	+3.14159	+0.32982

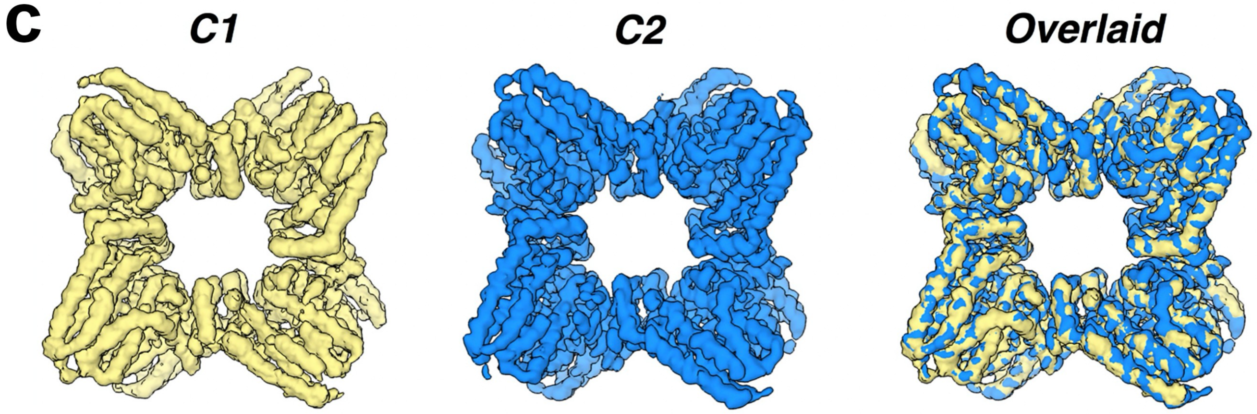

Supplementary Fig. 4. (a) Examples of density maps of the PaFS prenyltransferase. All maps are contoured at 0.0755 using Chimera, and local resolution ranges are indicated: **(i)** helix in chain A buried at the AB dimer interface; **(ii)** helix in chain B buried at the AB dimer interface; **(iii)** a solvent-exposed helix in chain A; **(iv)** a solvent-exposed helix in chain B. **(b)** Unbiased symmetry axis determination using proSHADE. The sharpened map of the PaFS transferase octamer solved with C1 symmetry to 4.18 Å resolution (Supplementary Fig. 3) was evaluated for unbiased symmetry axis determination using proSHADE. The most probable symmetry axis detected is indicated by a red arrow, corresponding to the parameters highlighted in boldface in the accompanying table. Weaker signals for other C2 and D2 symmetry axes are also identified. **(c)** Comparison of C1 and C2 reconstructions. A reconstruction of the PaFS octamer without symmetry expansion was resolved to 4.18 Å resolution (khaki), while refinement with C2 symmetry imposed yielded a reconstruction to 3.99 Å resolution (blue). Symmetric and asymmetric reconstructions can be superimposed with a correlation of 0.88 determined in Chimera. Maps contoured at 0.141 in Chimera.

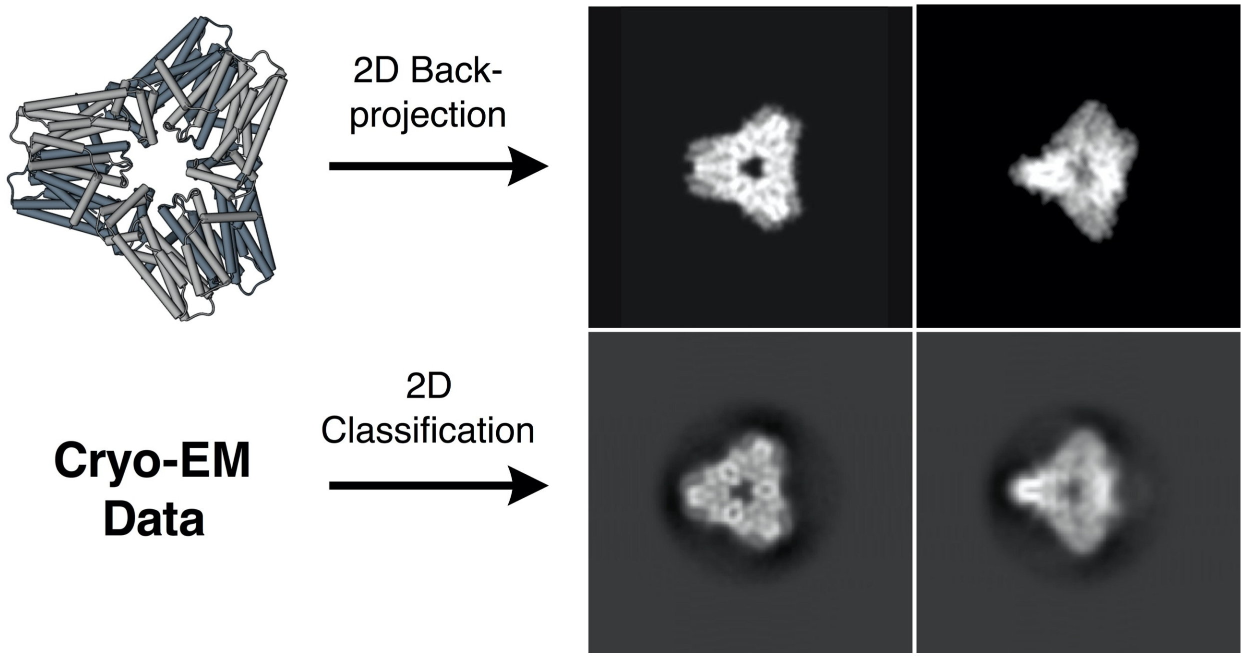

Supplementary Fig. 5. Crystal structure comparison with 2D class averages. The 2D back-projections of the PaFS prenyltransferase hexamer generated from the X-ray crystal structure (PDB 5ERN) low-pass filtered to 8 Å (top) display structural similarities with 2D class averages generated from cryo-EM experimental data (bottom). A satisfactory 3D reconstruction of the PaFS prenyltransferase hexamer from cryo-EM data could not be achieved due to an insufficient number of particles.

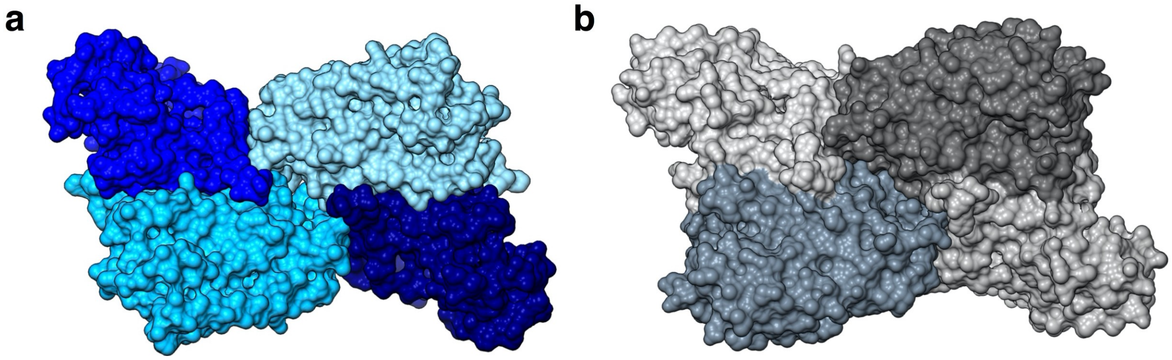

Supplementary Fig. 6. Comparison of side-views of octameric and hexameric PaFS prenyltransferases. (a) The interface of two dimers in the octamer, i.e., the tetramer of dimers, as observed in the cryo-EM structure. The cyan and dark blue monomers on the left comprise one AB dimer (listed as chain A and chain B in the atomic coordinates), and the blue and arctic blue monomers on the right comprise the A'B' dimer (listed as chain C and chain D, respectively, in the atomic coordinates) (PDB 7JTH). The AB-A'B' dimer-dimer interface has a buried surface area of 1012 $Å^2$; the other dimer-dimer interfaces (not shown) are similar but not identical with buried surface areas of 1013 $Å^2$, 1090 $Å^2$, and 1208 $Å^2$. **(b)** The corresponding dimer-dimer interface in the X-ray crystal structure of the hexamer (PDB 5ERN) is larger, with a total buried surface area of 1472 $Å^2$.

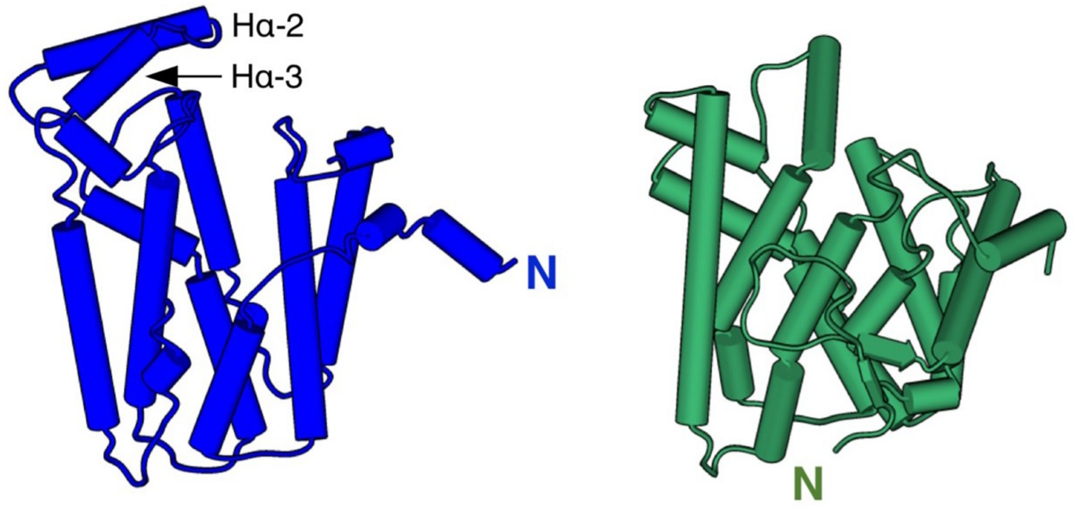

Supplementary Fig. 7. Comparison of the PaFS prenyltransferase and cyclase domains. Side-by-side comparison of the PaFS prenyltransferase (blue, PDB 5ERN) and cyclase (green, PDB 5ERM) domains highlights structural features that distinguish the two protein structures. In particular, the distinctive Hα-2 and Hα-3 helices that cap the active site of the prenyltransferase are absent in the cyclase.

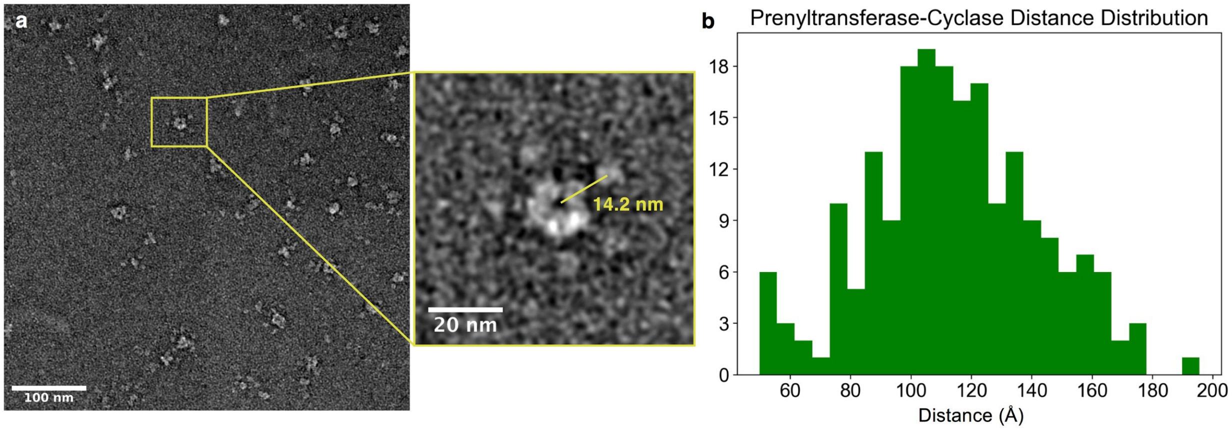

Supplementary Fig. 8. (a) Representative negative stain electron micrograph of PaFS with scale bar indicating 100 nm as shown in Supplementary Fig. 2a. Micrograph is representative of a dataset of 102 collected micrographs from one grid, and similar results were obtained from independent preparation of two other grids. Magnified region of micrograph displays a single particle with a representative distance measurement shown between the center of the octameric core and the center of a pendant cyclase domain; the scale bar indicates 20 nm. **(b)** Distribution of 202 prenyltransferase core–cyclase distances measured in negative stain electron micrographs. It was often difficult to distinguish prenyltransferase octamers from occasional hexamers in the micrographs due to low resolution. Measurements were made on prenyltransferases oriented such that the central pore was clearly visible; distances reported are from the center of the pore to the center of splayed-out cyclase domains, initially measured in pixels and then translated to Å. The average distance is 114 ± 28 Å (standard deviation); the minimum distance measured is 54 Å and the maximum distance measured is 196 Å. Source data are provided as a Source Data file.

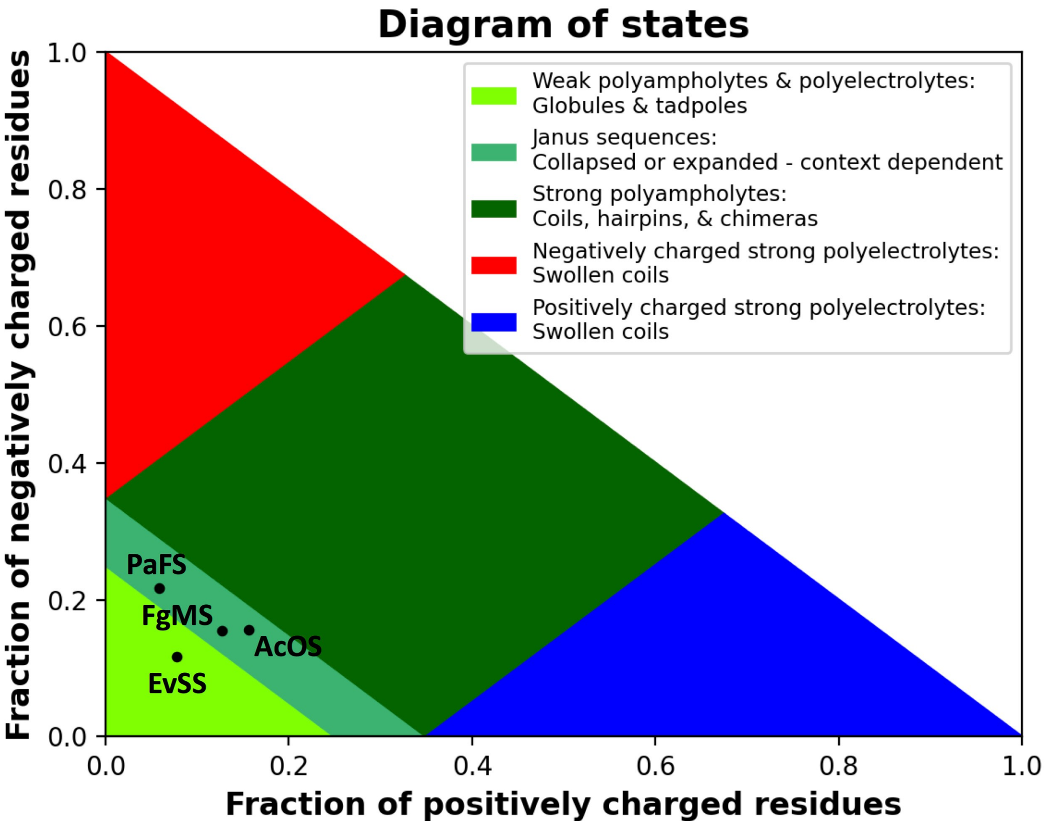

Supplementary Fig. 9. Structural classification of intrinsically disordered polypeptide linkers in bifunctional terpene synthases. The interdomain linkers in PaFS (fusicoccadiene synthase), FgMS (mangicdiene synthase), and AcOS (ophiobolin F synthase) are classified as Janus sequences that can adopt collapsed or expanded conformations depending on the context of the linkers. The interdomain linker in EvSS (stellatatriene synthase) is classified as a gobule. Sequence analyses were performed using localCIDER.[2]

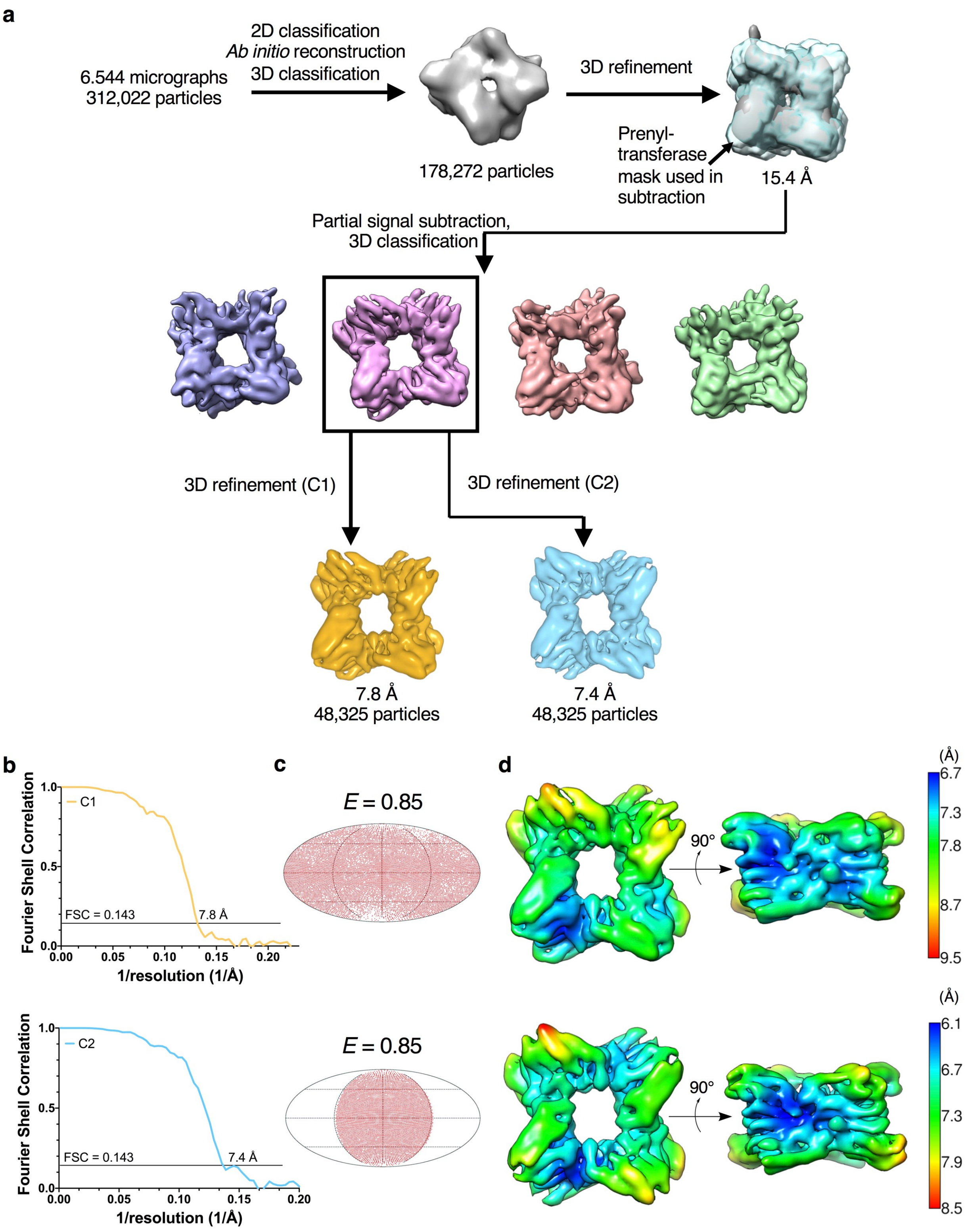

Supplementary Fig. 10. Workflow for cryo-EM structure determination of cross-linked PaFS prenyltransferase octamer. Particle picking was conducted using cryoSPARC; all other processing was performed with Relion. **(a)** Workflow of partial signal subtraction and masked classification used to obtain reconstruction of the PaFS octameric core. Partial signal subtraction was performed using the mask

shown surrounding the octameric core, where only density within the mask was retained. Masked 3D classification was conducted using the same mask used in subtraction. Particles from one selected class (48,325 particles) were refined with either C1 or C2 symmetry imposed to yield a reconstruction at 7.8 Å and 7.4 Å resolution, respectively. **(b)** FSC plots for maps calculated with C1 (top) and C2 (bottom) symmetry. Resolution estimates are indicated at 0.143 FSC. **(c)** Angular distribution spherical plots of data used in respective reconstructions. Efficiencies were calculated in cryo-EF and are listed as *E.* **(d)** Local resolution maps of respective reconstructions. Local resolution is depicted according to the scale shown in Å at 0.5 FSC. Maps calculated with Relion and contoured at 0.0529 (C1) and 0.0583 (C2) using Chimera.

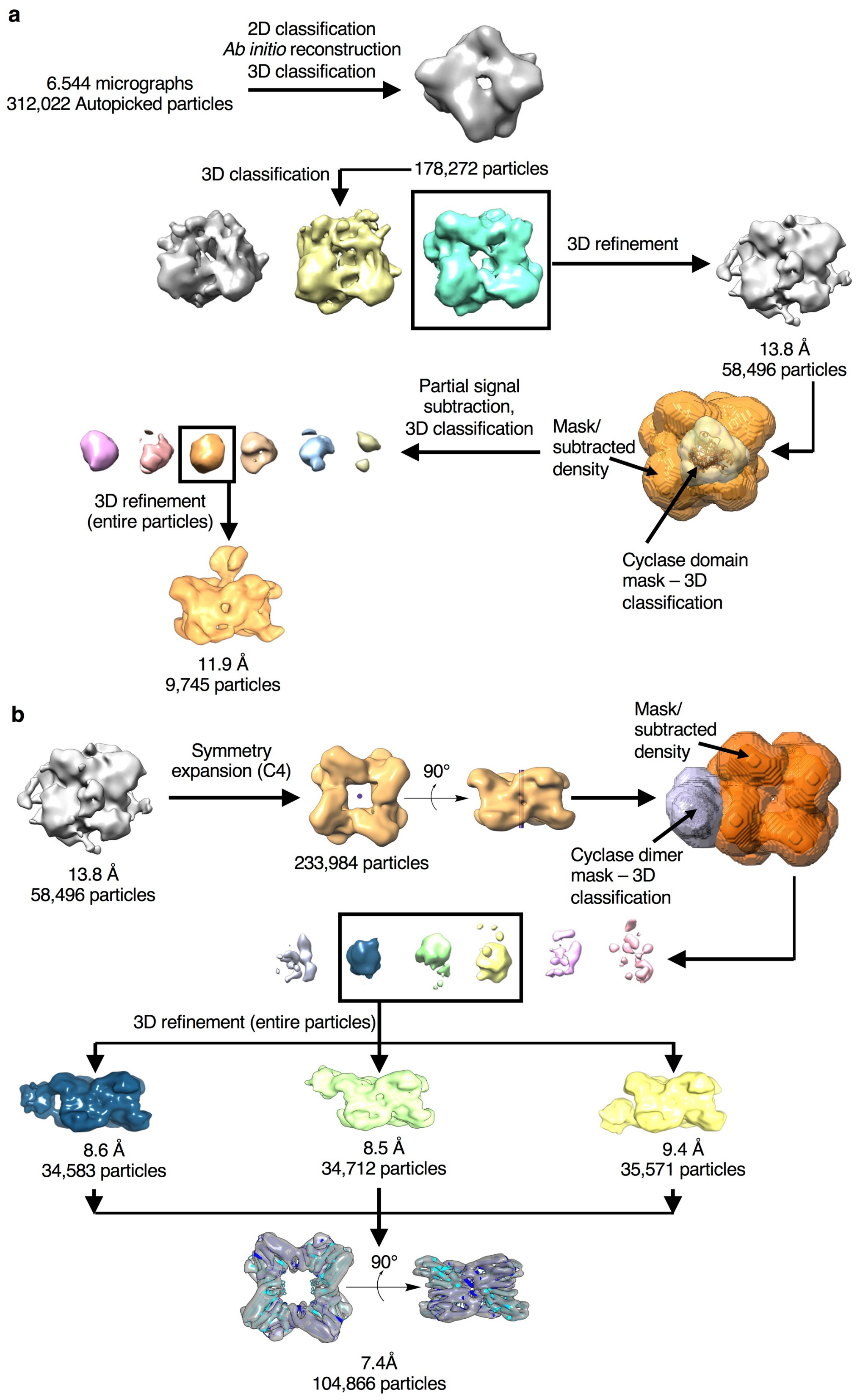
a
2D classification
Ab initio reconstruction
3D classification
6.544 micrographs
312,022 Autopicked particles
178,272 particles
3D classification
3D refinement
13.8 Å
58,496 particles
Partial signal
subtraction,
3D classification
Mask/
subtracted
density
Cyclase domain
mask – 3D
classification
3D refinement
(entire particles)
11.9 Å
9,745 particles
b
Symmetry
expansion (C4)
90°
Mask/
subtracted
density
13.8 Å
58,496 particles
233,984 particles
Cyclase dimer
mask – 3D
classification
3D refinement (entire particles)
8.6 Å
34,583 particles
8.5 Å
34,712 particles
9.4 Å
35,571 particles
90°
7.4Å
104,866 particles

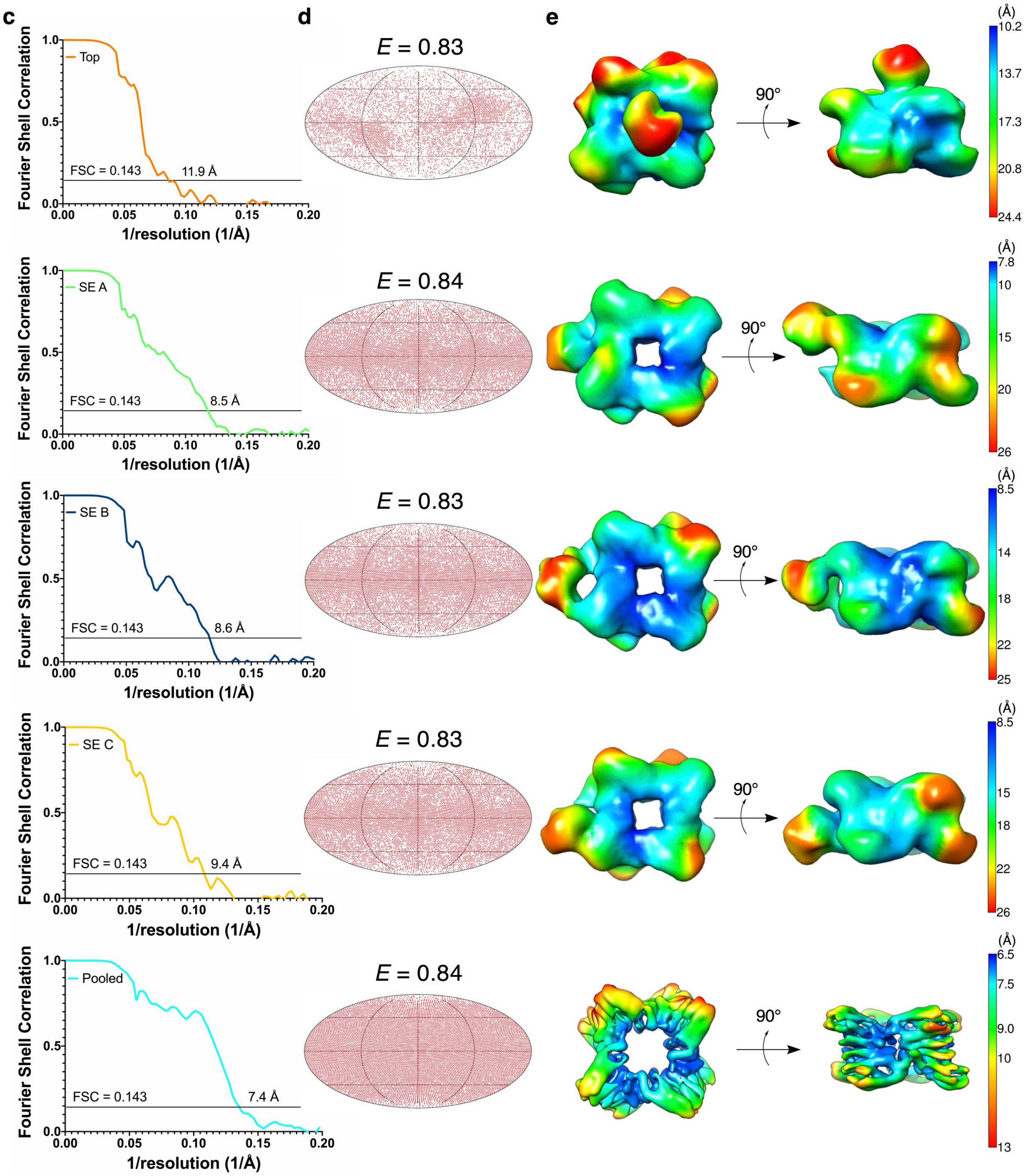

Supplementary Fig. 11. Workflow for cryo-EM structure determination of cross-linked PaFS. Particle picking was conducted using cryoSPARC; all other processing was performed with Relion. **(a)** Workflow of partial signal subtraction and masked classification used to obtain the reconstruction of the octameric prenyltransferase core of PaFS with a capping cyclase domain. After 3D classification and removal of junk particles, initial refinements of PaFS show converging density that capped the central pore. Partial signal subtraction was conducted using the mask shown surrounding the octameric core. Masked alignment free 3D classification was conducted using a mask calculated from the crystal structure of a cyclase domain (PDB 5ER8, chain A) modeled on top of the central pore. Entire particles from one

selected class (9,745 particles) were refined to yield a reconstruction of the prenyltransferase octamer with a cyclase domain capping the central pore. **(b)** Workflow of symmetry expansion, particle subtraction, and masked classification used to obtain reconstructions of the PaFS octameric core with peripheral cyclase domains. Particles were refined with imposed C4 symmetry, yielding the map shown; particles were then symmetry-expanded about the displayed C4 axis to yield 233,984 particles. Partial signal subtraction was then conducted using the mask shown surrounding the octameric core. Alignment free 3D classification was performed using a mask calculated from the crystal structure of the cyclase domain dimer (PDB 5ER8) modeled on the side of the central pore as shown. Entire particles from three selected classes were refined to yield symmetry-expanded (SE) reconstructions A, B, and C of the PaFS octameric core with the cyclase domain at three different positions, to 8.5 Å, 8.6 Å, and 9.4 Å resolution, respectively. Particles from these three classes were then pooled together, and further 3D refinement yielded reconstruction at 7.4 Å resolution. **(c)** FSC plots for the map containing the capping cyclase domain; SE maps A, B, and C; and the map of pooled SE particles. Resolution estimates are indicated at 0.143 FSC. **(d)** Angular distribution spherical plots of data used in respective reconstructions. Efficiencies were calculated in cryo-EF and are listed as *E*. **(e)** Local resolution maps of respective reconstructions. Local resolution is depicted according to the scale shown in Å at 0.5 FSC. Maps calculated with Relion and contoured at 0.0321 (top), 0.0354 (SE A), 0.0308 (SE B), 0.0274 (SE C), and 0.0718 (pooled SE) using Chimera.

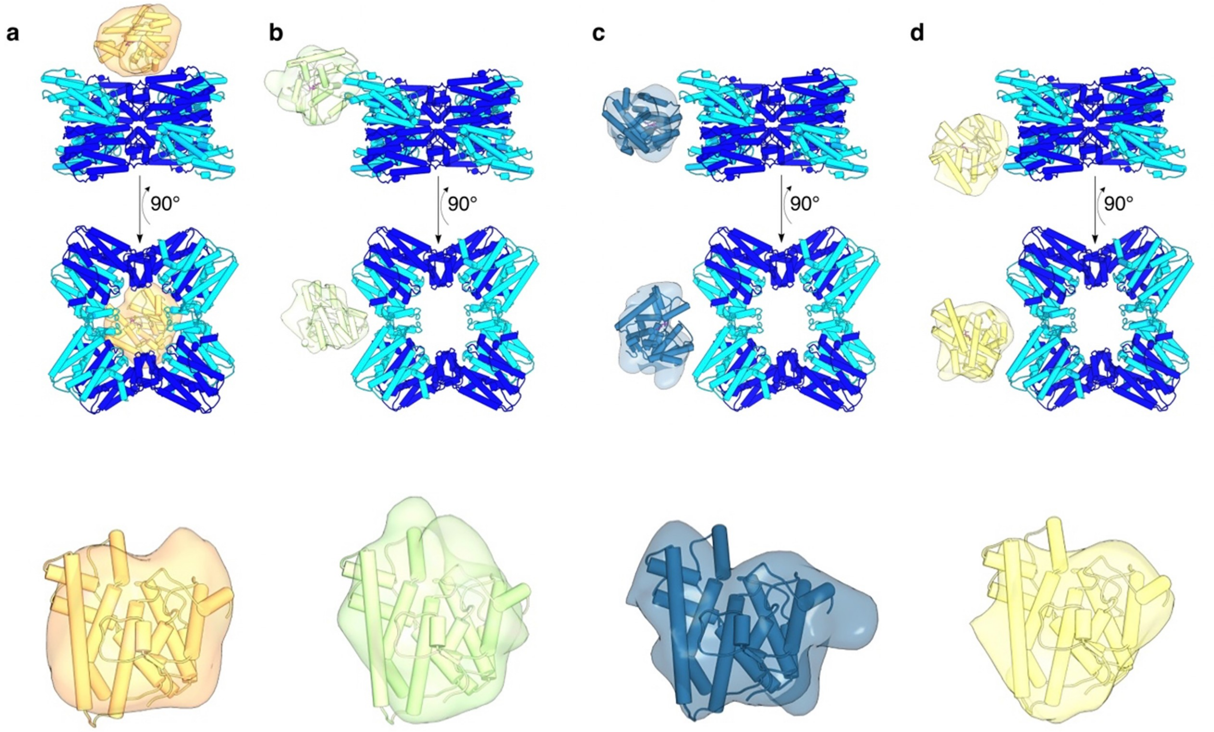

Supplementary Fig. 12. Variable positions and orientations of PaFS cyclase domains. Cryo-EM density of PaFS cyclase domains fit relative to the octameric prenyltransferase core. Density corresponding to each cyclase domain was tentatively fit with the crystal structure of the cyclase domain (PDB 5ER8, chain A, with bound ligand in the active site colored purple). Below each model is a close-up view of the cyclase domain fit into density, using a common orientation for each. **(a)** Density corresponding to one cyclase domain (contour 0.0191 in Chimera) caps the prenyltransferase octamer, modeled with the cyclase active site oriented toward the central pore. **(b)** Symmetry-expanded (SE) class A reveals density corresponding to one cyclase domain (contour 0.0132 in Chimera) positioned next to the prenyltransferase octamer. **(c)** SE class B shows density corresponding to one cyclase domain (contour 0.0172 in Chimera) positioned next to the prenyltransferase octamer, in a distinct position from classes A and C. **(d)** SE class C reveals density corresponding to one cyclase domain (contour in 0.0203 Chimera) positioned next to the prenyltransferase octamer, in a distinct position from classes A and B.

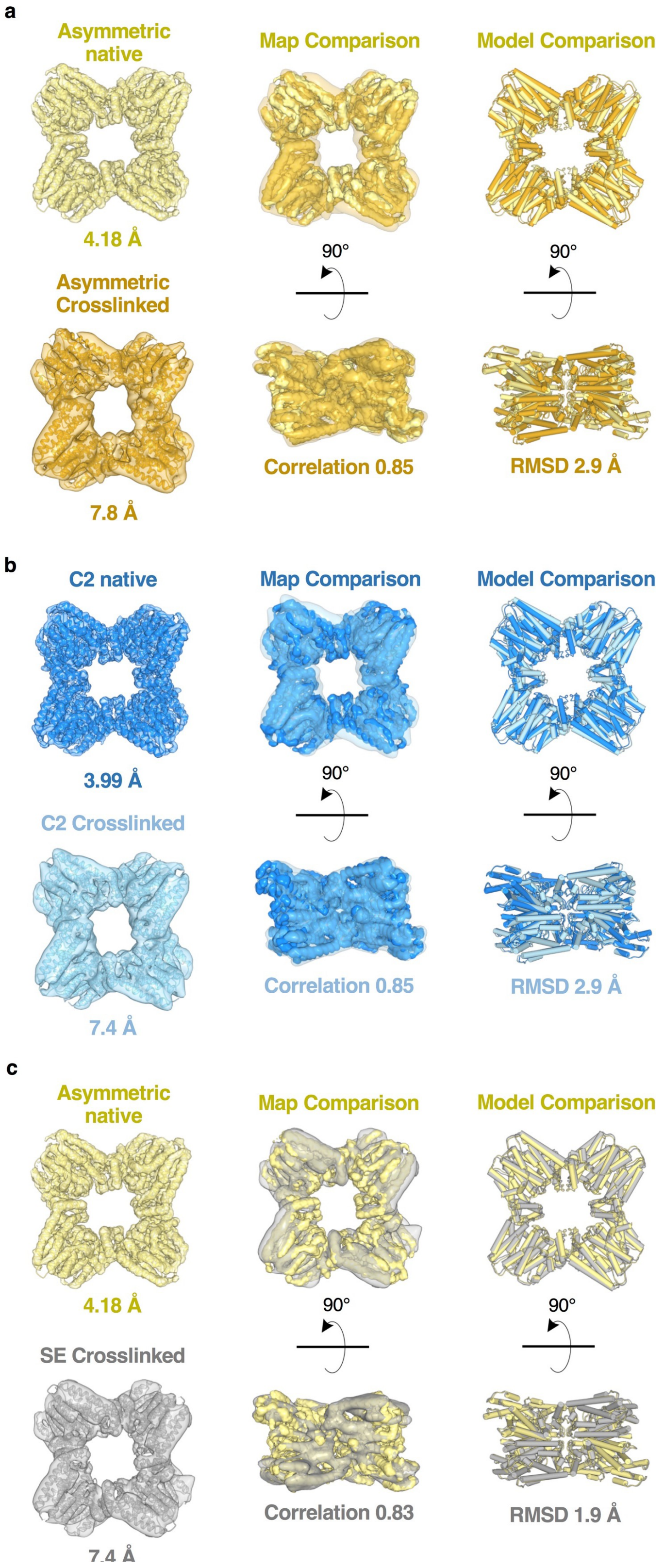
a
Asymmetric native
Map Comparison
Model Comparison
4.18 Å
90°
90°
Asymmetric Crosslinked
Correlation 0.85
RMSD 2.9 Å
7.8 Å
b
C2 native
Map Comparison
Model Comparison
3.99 Å
90°
90°
C2 Crosslinked
Correlation 0.85
RMSD 2.9 Å
7.4 Å
c
Asymmetric native
Map Comparison
Model Comparison
4.18 Å
90°
90°
SE Crosslinked
Correlation 0.83
RMSD 1.9 Å
7.4 Å

Supplementary Fig. 13. Fit of PaFS non-crosslinked octamer into crosslinked octamer density. (a) Comparison of asymmetric native reconstruction to asymmetric cross-linked reconstructions of the prenyltransferase core. Density for the octameric core in cross-linked PaFS (contour 0.0277 in Chimera) readily accommodates reconstruction of non-cross-linked PaFS (contour 0.141 in Chimera) with a correlation coefficient of 0.85. Atomic coordinates of the PaFS octamer fit into the cross-linked map chain-by-chain using Chimera yield an rmsd of 2.9 Å (2336 Cα atoms). **(b)** Comparison of C2-symmetric native reconstruction to C2-symmetric cross-linked reconstructions of prenyltransferase core. Density for the cross-linked octameric core (contour 0.0277 in Chimera) again readily accommodates density for native octameric core (contour 0.141 in Chimera) with 0.85 correlation. Atomic coordinates of the PaFS octamer fit into the cross-linked map chain-by-chain yield an rmsd of 2.9 Å (2336 Cα atoms). **(c)** Comparison of non-cross-linked, asymmetric prenyltransferase core and cross-linked, symmetry-expanded prenyltransferase core reconstructions. Consistent with results of reconstructions without symmetry expansion, density (contour 0.0395 in Chimera) readily accommodates that of non-cross-linked octameric core (0.83 correlation). When individual chains are fit into the density, comparison of native and crosslinked octamers yields an rmsd of 1.9 Å (2336 Cα atoms).

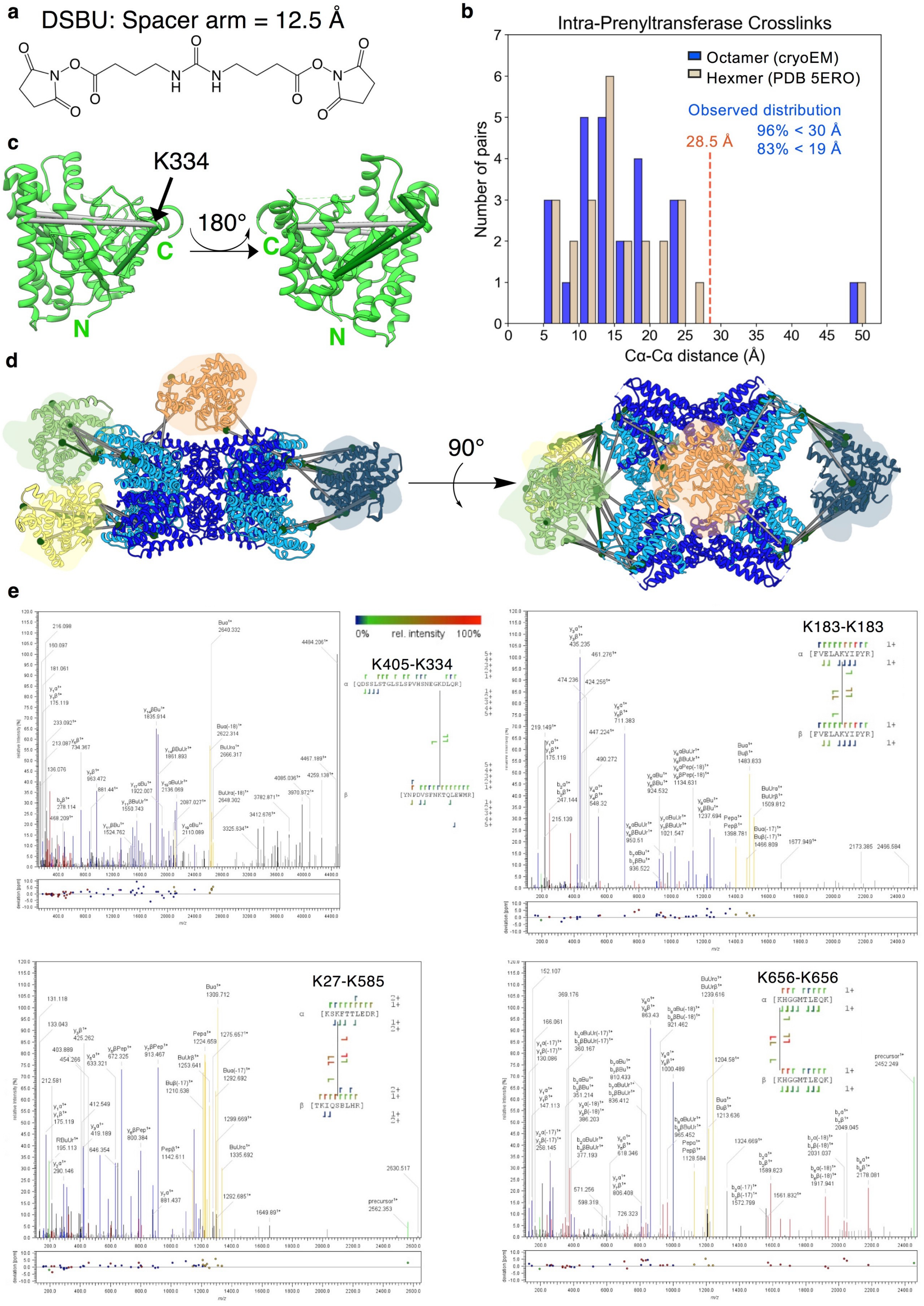
a
DSBU: Spacer arm = 12.5 Å
b
Intra-Prenyltransferase Crosslinks
Octamer (cryoEM)
Hexmer (PDB 5ERO)
Observed distribution
96% < 30 Å
83% < 19 Å
28.5 Å
Number of pairs
Cα-Cα distance (Å)
c
K334
180°
C
N
d
90°
e
K405-K334
K183-K183
K27-K585
K656-K656
0%
rel. intensity
100%

Supplementary Fig. 14. Crosslinking mass spectrometry (XL-MS). (a) Chemical structure of disuccinimidyl dibutyric urea (DSBU), the molecular crosslinker used for XL-MS studies. DSBU reacts with primary amines in lysine side chains as well as hydroxyl groups of serine, threonine, and tyrosine. These reactive groups must be within 12.5 Å, the length of the DSBU spacer arm. Adding twice the length of a lysine side chain (6-6.5 Å) plus estimated coordinate error for each mobile surface residue (1.5 Å) yields an approximate Cα-Cα distance cutoff of 28.5 Å. **(b)** Cα-Cα distance distribution of experimentally observed pairs in the PaFS prenyltransferase octamer (blue) and in the corresponding hexamer (tan). The approximate cross-link limit for DSBU of 28.5 Å is indicated by the dashed line; observed interactions falling below this limit (96% of observed pairs) are in agreement with the prenyltransferase octamer structure. **(c)** Intra-cyclase domain crosslinks modeled onto the crystal structure (PDB 5ERM, Chain A). Crosslinks within 30 Å are shown in dark green and longer than 30 Å in gray; such overlong crosslinks involve K334, which is adjacent to the flexible linker segment and may accordingly be more mobile in the full-length protein. **(d)** Model of the prenyltransferase octamer superimposed with the four cyclase domains positioned as shown in Fig. 3c. Selected interactions are modeled between cyclase and prenyltransferase domains with Cα atoms displayed as green spheres. Green crosslinks indicate Cα-Cα separations < 30 Å and gray crosslinks indicate Cα-Cα separations of 30–50 Å. All observed crosslinks are listed in Supplementary Table 3. **(e)** Spectra of selected crosslinks.

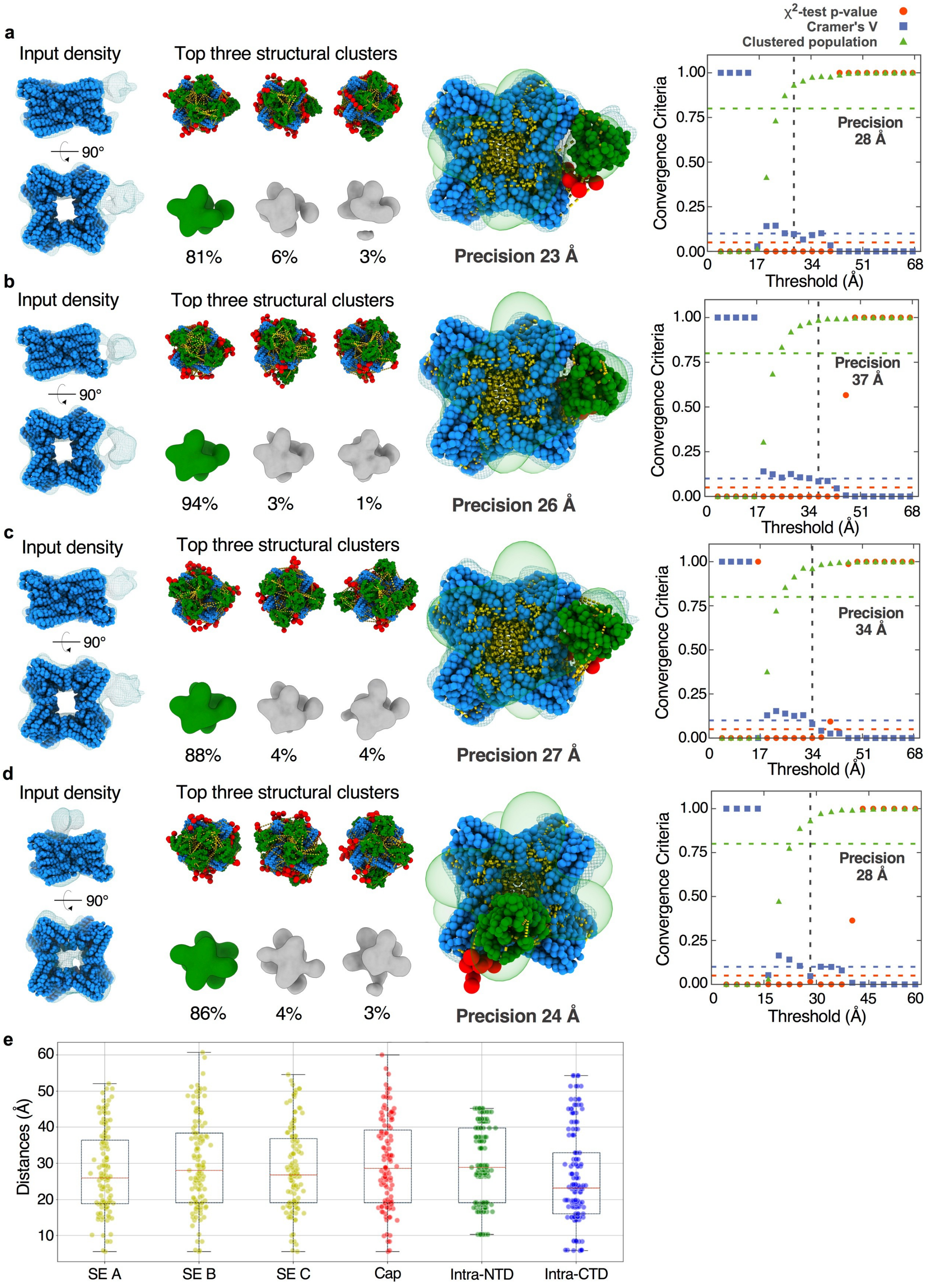

a
Input density
Top three structural clusters
90°
81%
6%
3%
Precision 23 Å
χ²-test p-value
Cramer's V
Clustered population
Convergence Criteria
Precision 28 Å
Threshold (Å)
b
Input density
Top three structural clusters
90°
94%
3%
1%
Precision 26 Å
Precision 37 Å
Threshold (Å)
c
Input density
Top three structural clusters
90°
88%
4%
4%
Precision 27 Å
Precision 34 Å
Threshold (Å)
d
Input density
Top three structural clusters
90°
86%
4%
3%
Precision 24 Å
Precision 28 Å
Threshold (Å)
e
Distances (Å)
SE A
SE B
SE C
Cap
Intra-NTD
Intra-CTD

Supplementary Fig. 15. Integrative Modeling of PaFS. (a–d) Input density, structural clusters and validations for simulations conducted using crosslinking data and low-resolution cryo-EM maps of the Symmetry Expanded (SE) class A **(a)**, SE class B **(b)**, SE class C **(c)** and capping domain **(d)**. Input density displayed as Gaussian mixture model (GMM) with atomic coordinates of octameric prenyltransferase domain (PDB 7JTH) shown as one residue per bead. Simulations utilizing GMMs and crosslinking data resulted in 7–15 converging structural clusters (Table S4) with the top three structural clusters shown here as localization probability densities with their population percentages. The top structural cluster from each simulation is displayed with octameric prenyltransferase core (blue) and cyclase domain (green) modeled at one residue per bead and linker segment (red) shown as 10 residues per bead (red). Crosslinks are displayed as yellow dashed lines. Convergence criteria plots are shown for each simulation, with precisions reported on the graph (right). Source data are provided as a Source Data file. **(e)** Each box and whiskers plot represents the distributions of distances of observed crosslinked residue pairs from the dominant structural model in each SE or capping domain class. Crosslinks beyond 55 Å were excluded for the intra-CTD model as these were observed to be intermolecular crosslinks between distant monomers. The median modeled crosslink distance is shown as a red line in the box, with the ends of each box representing the lower and upper quartiles within the distribution of distances modeled. Whiskers and lines represent the range of the modeled distances. For all plates, source data are provided as a Source Data file.

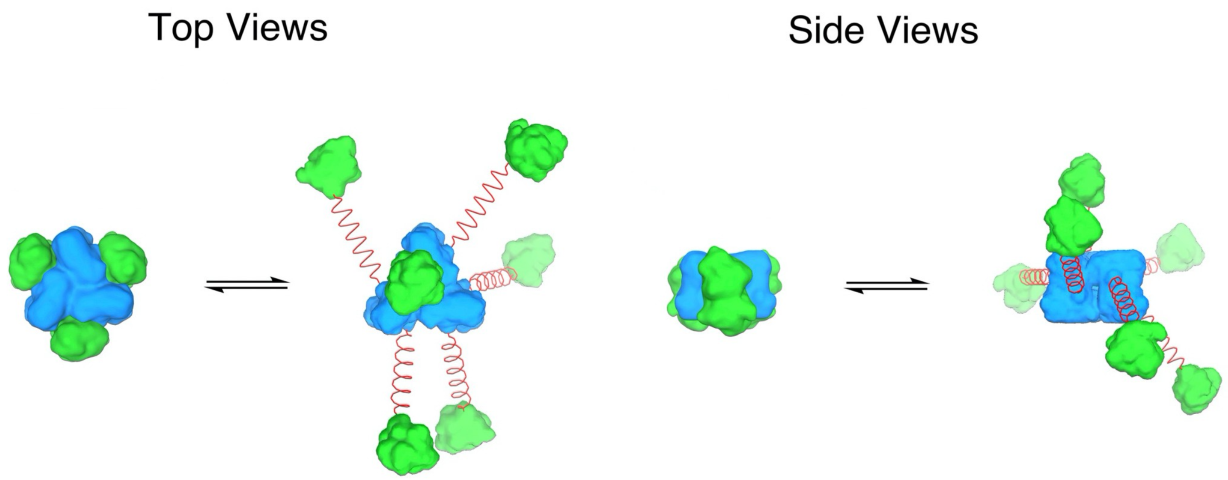

Supplementary Fig. 16. Possible conformations of the full-length PaFS hexamer. Prenyltransferase domains (blue) and cyclase domains (green) are represented by molecular surfaces generated with UCSF Chimera; the flexible 70-residue linker is represented by a red spring (this color scheme corresponds to the primary structure summary in Fig. 1). The compact hexamer conformation derives from the molecular envelope of N333A/Q336A PaFS calculated from small-angle X-ray scattering (SAXS) data[3]. Cyclase domains in splayed-out positions are modeled with an average distance of 120 Å from the prenyltransferase core.

Supplementary References

1. Ronnebaum, T. A., Gupta, K. & Christianson, D. W. Higher-order oligomerization of a chimeric αβɣ bifunctional diterpene synthase with prenyltransferase and class II cyclase activities is concentration-dependent. *J. Struct. Biol.* **210**, 107463 (2020).

2. Holehouse, A. S., Das, R. K., Ahad, J. N., Richardson, M. O. G. & Pappu, R. V. CIDER: Resources to analyze sequence-ensemble relationships of intrinsically disordered proteins. *Biophys. J.* **112**, 16–21 (2017).

3. Chen, M., Chou, W. K. W., Toyomasu, T., Cane, D. E. & Christianson, D. W. Structure and function of fusicoccadiene synthase, a hexameric bifunctional diterpene synthase. *ACS Chem. Biol.* **11**, 889–899 (2016).
