# Supplementary material for: Structural insight on assembly-line catalysis in terpene biosynthesis
Source: Nat Commun. 2021 Jun 9;12:3487. doi: 10.1038/s41467-021-23589-9 (PMC8190136; doi:10.1038/s41467-021-23589-9)
Supplement: Supplementary file 2 — Description of Additional Supplementary Files [file 41467_2021_23589_MOESM2_ESM.docx]

**Description of Additional Supplementary Files**

**File Name:** Supplementary Data 1

**Description:** Raw MeroX results table.
