# Supplementary material for: Structural insight on assembly-line catalysis in terpene biosynthesis
Source: Nat Commun. 2021 Jun 9;12:3487. doi: 10.1038/s41467-021-23589-9 (PMC8190136; doi:10.1038/s41467-021-23589-9)
Supplement: Supplementary file 4 — Reporting Summary [file 41467_2021_23589_MOESM4_ESM.pdf]

nature research

Corresponding author(s): David Christianson
Last updated by author(s): Apr 29, 2021

Reporting Summary

Nature Research wishes to improve the reproducibility of the work that we publish. This form provides structure for consistency and transparency in reporting. For further information on Nature Research policies, see our Editorial Policies and the Editorial Policy Checklist.

Statistics

For all statistical analyses, confirm that the following items are present in the figure legend, table legend, main text, or Methods section.

n/a	Confirmed	
☐	☒	The exact sample size (n) for each experimental group/condition, given as a discrete number and unit of measurement
☐	☒	A statement on whether measurements were taken from distinct samples or whether the same sample was measured repeatedly
☐	☒	The statistical test(s) used AND whether they are one- or two-sided<br>*Only common tests should be described solely by name; describe more complex techniques in the Methods section.*
☒	☐	A description of all covariates tested
☒	☐	A description of any assumptions or corrections, such as tests of normality and adjustment for multiple comparisons
☐	☒	A full description of the statistical parameters including central tendency (e.g. means) or other basic estimates (e.g. regression coefficient) AND variation (e.g. standard deviation) or associated estimates of uncertainty (e.g. confidence intervals)
☐	☒	For null hypothesis testing, the test statistic (e.g. F, t, r) with confidence intervals, effect sizes, degrees of freedom and P value noted<br>*Give P values as exact values whenever suitable.*
☒	☐	For Bayesian analysis, information on the choice of priors and Markov chain Monte Carlo settings
☒	☐	For hierarchical and complex designs, identification of the appropriate level for tests and full reporting of outcomes
☒	☐	Estimates of effect sizes (e.g. Cohen's d, Pearson's r), indicating how they were calculated

Our web collection on statistics for biologists contains articles on many of the points above.

Software and code

Policy information about availability of computer code

Data collection	SerialEM (v3.8) was used for cryo-EM data collection of unfixed PaFS; Latitude (Gatan) was used for cryo-EM data collection of Grafix-stabilized PaFS.
Data analysis	EMAN (2.31) suite, RELION (v2.1), and CTFFIND (v4.1) were used to analyze negative stain EM data. To analyze unfixed PaFS cryo-EM data, cryoSPARC (v2.5.1), RELION (v3.0.7), CTFFIND (v4.1), and DeepEMhancer (v2.0) were used, as well as Phenix (dev-3736), Coot (v 0.8.9.1), and MolProbity (v4.5.1) for model refinement and validation; to analyze Grafix-stabilized PaFS cryo-EM data, cryoSPARC (v2.15), RELION (v3.0.7), and Phenix (dev-3736) were used. Cryo-EF (v1.1) was used for reconstruction validation; proSHADE (v0.7.5.0) was used for unbiased symmetry determination; Prism Graphpad (v9.0.0) was used for FSC curve visualization. localCIDER (v0.1.18) was used for analysis of disordered linker sequences. TurboRawtoMGF (v2.0.8) was used to convert raw crosslinking-mass spectrometry files and MeroX (v2.0.1.4) was used to analyze XL-MS data. For visualization of all cryo-EM and XL-MS data, Chimera (v1.13.1) was used. Integrative Modeling Platform (IMP) (v2.14.0) was used for integrative modeling, and ChimeraX (v1.1) was used for visualization. Data for the entire integrative modeling project are accessible on Github: https://github.com/cryomurakami/Structural-Insight-on-Assembly-Line-Catalysis-in-TerpeneBiosynthesis

For manuscripts utilizing custom algorithms or software that are central to the research but not yet described in published literature, software must be made available to editors and reviewers. We strongly encourage code deposition in a community repository (e.g. GitHub). See the Nature Research guidelines for submitting code & software for further information.

Data

Policy information about availability of data

All manuscripts must include a data availability statement. This statement should provide the following information, where applicable:

- Accession codes, unique identifiers, or web links for publicly available datasets
- A list of figures that have associated raw data
- A description of any restrictions on data availability

Atomic coordinates of the PaFS prenyltransferase octamer (C2 symmetry) have been deposited in the Protein Data Bank (www.rcsb.org) with PDB accession code 7JTH [https:// doi.org/10.2210/pdb7JTH/pdb], and in the Electron Microscopy Data Bank (EMDB, https://www.ebi.ac.uk/pdbe/emdb) with accession code EMD-22473 [https://www.ebi.ac.uk/pdbe/entry/emdb/EMD-22473]. The asymmetric (C1) density map has also been deposited in the EMDB with accession code EMD-23602 [https://www.ebi.ac.uk/pdbe/entry/emdb/EMD-23602]. Additionally, density maps of the crosslinked PaFS prenyltransferase octamer have been deposited in the EMDB with accession codes EMD-23610 [https://www.ebi.ac.uk/pdbe/entry/emdb/EMD-23610] (C1) and EMD-23611 [https://www.ebi.ac.uk/pdbe/entry/emdb/EMD-23611] (C2). Density maps of the fixed PaFS prenyltransferase octamer with associated cyclase domains have been deposited in the EMDB with accession codes as follows: capping cyclase domain, EMD-22489 [https://www.ebi.ac.uk/pdbe/entry/emdb/EMD-22489]; Symmetry Expanded (SE) Class A domain, EMD-22485 [https://www.ebi.ac.uk/pdbe/entry/emdb/EMD-22485]; SE Class B domain, EMD-22487 [https://www.ebi.ac.uk/pdbe/entry/emdb/EMD-22487]; SE Class C domain, EMD-22488 [https://www.ebi.ac.uk/pdbe/entry/emdb/EMD-22488]; Pooled SE Domain Classes A–C, EMD-22484 [https://www.ebi.ac.uk/pdbe/entry/emdb/EMD-22484]. The common Repository of Adventitious Proteins (cRAP) database used in this study can be accessed at https://www.thegpm.org/crap. Crosslinking-mass spectrometry data have been deposited in the PRIDE repository with dataset identifier PRIDE: PXD021007 [http://proteomecentral.proteomexchange.org/cgi/GetDataset?ID=PXD021007]. Previously determined crystal structures of the individual PaFS prenyltransferase and cyclase domains were also used in this study and can be accessed in the PDB with accession codes 5ERN [https://doi.org/10.2210/pdb5ERN/pdb] and 5ERM [https://doi.org/10.2210/pdb5ERM/pdb] or 5ER8 [https://doi.org/10.2210/pdb5ER8/pdb], respectively. Source data are provided with this paper. All other relevant data are available from the corresponding author upon request.

Field-specific reporting

Please select the one below that is the best fit for your research. If you are not sure, read the appropriate sections before making your selection.

[x] Life sciences [ ] Behavioural & social sciences [ ] Ecological, evolutionary & environmental sciences

For a reference copy of the document with all sections, see nature.com/documents/nr-reporting-summary-flat.pdf

Life sciences study design

All studies must disclose on these points even when the disclosure is negative.

Sample size	For kinetic studies, sample size was determined based on standards in the field, attempting to have a minimum N = 3 with sufficient reproducibility.
Data exclusions	N/A
Replication	Kinetic assays were each conducted three times independently with similar results. An SDS-PAGE of PaFS at room temperature over time was run once; PaFS was independently run on SDS-PAGE more than ten times, with consistent and reproducible results. Native-PAGE of PaFS was independently conducted three times with similar results. All replications were successful. For negative stain electron microscopy, three grids were independently prepared and examined; separate datasets were collected from each grid with similar results obtained, but only the dataset (containing 102 micrographs) with highest quality particle distribution was used for analysis. For cryo-electron microscopy several grids were screened for data collection but only the grids with the highest quality ice and particle distribution were used for data collection.
Randomization	N/A
Blinding	N/A

Reporting for specific materials, systems and methods

We require information from authors about some types of materials, experimental systems and methods used in many studies. Here, indicate whether each material, system or method listed is relevant to your study. If you are not sure if a list item applies to your research, read the appropriate section before selecting a response.

Materials & experimental systems

n/a	Involved in the study
☒	☐ Antibodies
☒	☐ Eukaryotic cell lines
☒	☐ Palaeontology and archaeology
☒	☐ Animals and other organisms
☒	☐ Human research participants
☒	☐ Clinical data
☒	☐ Dual use research of concern

Methods

n/a	Involved in the study
☒	☐ ChIP-seq
☒	☐ Flow cytometry
☒	☐ MRI-based neuroimaging
